# Early Onset Pre-Eclampsia Is Associated with Altered DNA Methylation of Cortisol-Signalling and Steroidogenic Genes in the Placenta

**DOI:** 10.1371/journal.pone.0062969

**Published:** 2013-05-07

**Authors:** Kirsten Hogg, John D. Blair, Deborah E. McFadden, Peter von Dadelszen, Wendy P. Robinson

**Affiliations:** 1 Department of Medical Genetics, University of British Columbia, Vancouver, British Columbia, Canada; 2 Department of Pathology, University of British Columbia, Vancouver, British Columbia, Canada; 3 Department of Obstetrics and Gynaecology, University of British Columbia, Vancouver, British Columbia, Canada; 4 Child & Family Research Institute, Vancouver, British Columbia, Canada; VU University Medical Center, The Netherlands

## Abstract

Placental cortisol is inactivated in normotensive pregnancies, but is frequently present in pre-eclampsia associated placentae. Since glucocorticoids are strongly associated with the programming of long-term health, we assessed DNA methylation of genes involved in cortisol signalling and bioavailability, and hormonal signalling in the placenta of normotensive and hypertensive pregnancies. Candidate genes/CpG sites were selected through analysis of Illumina Infinium HumanMethylation450 BeadChip array data on control (n = 19) and early onset pre-eclampsia (EOPET; n = 19) placental samples. DNA methylation was further quantified by bisulfite pyrosequencing in a larger cohort of control (n = 111) cases, in addition to EOPET (n = 19), late onset pre-eclampsia (LOPET; n = 18) and normotensive intrauterine growth restriction (nIUGR; n = 13) cases. DNA methylation (percentage points) was increased at CpG sites within genes encoding the glucocorticoid receptor (*NR3C1* exon 1D promoter; +8.46%; *P*<0.01) and corticotropin releasing hormone (CRH) binding protein (*CRHBP* intron 3; +9.14%; *P*<0.05), and decreased within *CRH* (5′ UTR; −4.30%; *P* = 0.11) in EOPET-associated placentae, but not in LOPET nor nIUGR cases, compared to controls. Differential DNA methylation was not observed among groups at the 11β-hydroxysteroid dehydrogenase type 2 (*HSD11B2*) gene promoter. Significant hypomethylation was observed in pre-eclampsia but not nIUGR placentae for steroidogenic genes, including *CYP11A1* (exon1; EOPET; −9.66%; *P*<0.00001, and LOPET; −5.77%; *P*<0.001), 3β-hydroxy-delta-5-steroid dehydrogenase type 1 (*HSD3B1* exon 2; EOPET; −12.49%; *P*<0.00001, and LOPET; −6.88%; *P*<0.001), TEA domain family member 3 (*TEAD3* intron 1; EOPET; −12.56%; *P*<0.00001) and *CYP19* (placental-specific exon 1.1 promoter; EOPET; −10.62%, *P*<0.0001). These data represent dysregulation of the placental epigenome in pre-eclampsia related to genes involved in maintaining the hormonal environment during pregnancy and highlights particular susceptibility in the early onset syndrome.

## Introduction

Maternal stress and/or elevated glucocorticoid exposure *in utero* is associated with low birth weight, exaggerated infant stress response and adult disease in the offspring [1]. Disorders during pregnancy which alter maternal physiology and induce systemic inflammatory response are likely to augment stress-signalling pathways, and may be associated with adverse developmental programming. Pre-eclampsia, particularly that of early onset, is largely the result of poor placentation, leading to hypoxic and inflammatory insult to surrounding tissue as well as under-perfusion of essential materials from mother to fetus [2]–[4]. Further maternal complications can include necrosis of the liver, respiratory distress syndrome and seizures of eclampsia [5]. Clinically, pre-eclampsia is a common obstetrical complication in developed countries and remains a leading cause of maternal death [6], however, its aetiology is poorly understood. In this study we aim to determine how placental stress-pathways might be altered in pre-eclampsia and related pregnancy complications.

Cortisol is responsible for the physiological ‘stress response’, mediated through binding to the glucocorticoid receptor (GR) gene (nuclear receptor subfamily 3, group C, member 1 [*NR3C1*]) [7]. The release of cortisol may be triggered by a number of stimulatory cues to regulate immune response, cardiovascular function, metabolism and reproductive function [8]. Corticotropin releasing hormone (CRH) initiates this stress response through the hypothalamic pituitary adrenal (HPA) axis. Large quantities of placental CRH, identical to the hypothalamic form, are synthesised and secreted by the syncytiotrophoblast [9], [10] and stimulate both the maternal and fetal pituitary to release adrenocorticotropin hormone (ACTH), thus inducing adrenal cortisol secretion [11]–[13]. In the placenta, CRH has multiple roles including the stimulation of estrogen synthesis [14] and increasing the availability of maternal glucose for placental uptake [15]. In contrast to hypothalamic CRH control, cortisol positively stimulates CRH production in the placenta [16], [17]. CRH activity is controlled by sequestration to plasma CRH binding protein (CRHBP), thus regulating the levels of bioactive free CRH [13].

In the placenta, cortisol is inactivated by 11β-hydroxysteroid dehydrogenase type 2 (11β-HSD2), encoded for by *HSD11B2* [18]. Dysregulation of *HSD11B2* in the placenta is linked to the detrimental actions of cortisol in offspring and is accompanied by low birth weight [19]–[22]. Pregnancy complications, including pre-eclampsia [23], [24] and intrauterine growth restriction (IUGR) [25]–[27] have been associated with reduced placental activity/expression of *HSD11B2*.

Epigenetics is commonly studied in the context of developmental programming and DNA methylation is the most studied epigenetic mark [28]. The placenta largely contributes to the fetal environment through its endocrine and metabolic functions, in addition to nutrient exchange. While this alone can influence epigenetic susceptibility in the fetus, the placenta is also a target of environmental effects (reviewed in [29]). Illustrative of potential placental susceptibility to stress *in utero*, Wyrwoll *et al*. recently reported altered methyl donor components, including folate, methionine and choline in the placenta of rats exposed to glucocorticoids [30].

DNA methylation of the *NR3C1* exon 1F promoter is widely studied, where altered methylation is observed in response to stress stimuli in rat [31] and human [32]–[37] studies across multiple tissues. DNA methylation is also proposed to regulate tissue-specific *HSD11B2* expression [38], [39]; and has been associated with placental *HSD11B2* gene regulation in prenatally stress-exposed rats [40]. In addition, *CRH* mRNA expression in the human placenta is susceptible to increased maternal intake of the methyl donor choline, which is associated with increased DNA methylation of the *CRH* promoter [41].

The aim of this study was to assess the impact of placental pathology, including pre-eclampsia and normotensive (n)IUGR cases, on the DNA methylation status of several genes that are linked to stress-induced pathways. For this purpose, pre-eclampsia was further subdivided into two categories: those patients with early onset symptoms arising <34^+0^ weeks gestation (early onset pre-eclampsia [EOPET]), or arising ≥34^+0^ weeks gestation (late onset pre-eclampsia [LOPET]), the former of which carries a considerably worse maternal and fetal prognosis [5]. A candidate gene approach was undertaken through analysis of Illumina Infinium HumanMethylation450 BeadChip array data targeting >450,000 CpG sites across the human genome in a control and EOPET subset, followed by confirmation and extension to additional cohorts using bisulfite pyrosequencing.

Genes selected for analysis included those involved in 1) cortisol signalling: *NR3C1*, *CRH*, and CRH receptor (*CRHR1*) and pro-opiomelanocortin (*POMC* [ACTH precursor]), 2) cortisol bioavailability: *CRHBP* and *HSD11B2*, and 3) placental hormonal signalling: StAR-related lipid transfer domain protein 3 (*STARD3*), *CYP11A1*, 3β-hydroxy-delta-5-steroid dehydrogenase type 1 (*HSD3B1*), TEA domain family member 3 (*TEAD3*) and *CYP19*. Herein, we show the remarkable DNA methylation plasticity of many genes involved in stress and hormone pathways associated with the severe placental dysfunction present in EOPET.

## Materials and Methods

### Ethics Statement

This study was approved by the University of British Columbia and the Children’s & Women’s Health Centre of British Columbia. Women between the ages of 18 and 42 years were recruited from the BC Women & Children’s Hospital, Vancouver, during the second trimester of pregnancy following informed written consent.

### Subjects

Cases included in this study partially overlap other studies published by our group [42]–[45]. Control cases consisted of normotensive women with no known placental pathology. In order to collect control samples at gestational ages matching that of case cohorts, premature deliveries involving a range of causes such as premature rupture of membranes, chorioamnionitis and cervical incompetence were included. Pre-eclampsia was defined using the Society of Obstetricians and Gynaecologists of Canada guidelines [46] which include the following criteria: 1) hypertension (systolic blood pressure ≥140 mmHg and/or diastolic blood pressure ≥90 mmHg measured twice, at least 4 hours apart) after 20 weeks gestation in combination with proteinuria (≥0.3 g/d or ≥2+ dipstick proteinuria) or another adverse condition (non-hypertensive and non-proteinuric haemolysis, elevated liver enzymes, low platelet count (HELLP) syndrome, fetal IUGR; or absent or reversed end-diastolic flow in the umbilical artery by Doppler velocimetry). EOPET was defined as symptoms arising <34^+0^ weeks and LOPET as symptoms arising ≥34^+0^ weeks gestation [5]. nIUGR was defined as either: birth weight below the 3^rd^ percentile for gender and gestational age using Canadian population parameters [47], or birth weight below the 10^th^ percentile in combination with a) persistent uterine artery notching at 22^+0^ to 24^+6^ weeks, b) absent or reversed end diastolic velocity on umbilical artery Doppler, and/or c) oligohydramnios (amniotic fluid index <50 mm) and absent maternal hypertension. Gestational diabetes, isolated maternal hypertension, non-singleton pregnancies, still birth or fetal genetic anomaly cases were excluded from the study.

Patient characteristics for the full cohort are outlined in Table 1. As expected, birth weight was significantly lower in all case groups compared to controls. Furthermore, IUGR co-existed with pre-eclampsia in 14/19 EOPET and 5/18 LOPET cases. Gestational age was not matched in pre-eclampsia and control cases and was assessed as a confounder in DNA methylation studies. Where possible, births were defined by the presence (spontaneous or induced vaginal delivery) or absence (elective caesarean section) of labour (Table 1). Emergency caesareans, or cases in which no data were available, were undefined. Labour was therefore defined in 54/111 controls, 10/19 EOPET, 12/18 LOPET and 9/13 nIUGR patients. Of these cases there was unequal distribution of labour/no labour births in EOPET and nIUGR patients compared with controls (χ^2^; *P*<0.001, *P<*0.05, respectively). Self-reported ethnicity was available in only a minority of cases, precluding evaluation of this as a separate variable. Of these, the majority reported Caucasian (∼60%) or Asian (∼30%), with the remainder of other or mixed ancestry.

**Table 1:** Patient characteristics for cases assessed by bisulfite pyrosequencing.

Parameter	Controls n = 111	EOPET n = 19	LOPET n = 18	nIUGR n = 13
Birth weight (g)	2590±989	1399±621	2742±798	2132±513
Birth weight (SD)	0.06±0.07	−1.51±0.22***	−0.73±0.29**	−1.67±0.20***
Co-existing IUGR	–	14/19	5/18	–
Gestational age (weeks)	35.09±4.21	31.93±3.27*	37.47±2.29*	36.43±2.31
Maternal age (years)	33.10±4.74	34.16±6.01	33.49±5.47	34.64±5.26
Female: Male ratio	55∶56	7∶12	10∶8	9∶4
Mode of delivery				
Labour	46	4	9	5
No Labour	8	6	3	4
Undefined	57	9	6	4

Birth weight statistics are based on standard deviation (SD) points relative to gestational age and gender matched normal birth weight ranges. Gestational age values are given at birth. Data are mean ± SD. The mode of delivery is characterised as ‘Labour’ (spontaneous or induced vaginal delivery), ‘No Labour’ (elective caesarean section) or, ‘Undefined’: emergency caesarean section or unknown. EOPET: early onset pre-eclampsia, LOPET: late onset pre-eclampsia, nIUGR: normotensive intrauterine growth restriction,

* *P*<0.05,

** *P*<0.01,

*** *P*<0.001, compared to controls.

### Selection of Candidate Genes

A candidate gene approach was used to select a set of genes known to be directly involved in stress hormone signalling, availability and downstream actions, such as hormone production. Data were collected from an Infinium HumanMethylation450 BeadChip array (Illumina Inc., San Diego, CA); genome-wide results are being published separately by our group (Blair *et al*., in preparation; GEO: GSE44667). Briefly, tissue (1 cm^3^) was sampled from three chorionic villus sites and DNA extracted using standard salting out methods. Equal amounts of placental DNA were pooled from all three sites per placenta and bisulfite converted using the EZ DNA methylation kit (Zymo Research Corp., Irvine, CA) following the manufacturer’s instructions. A subset of gestational age and gender matched control (n = 19) and EOPET (n = 19) placental DNA samples were run on the Infinium HumanMethylation450 BeadChip array according to the manufacturer’s protocol. Raw data were read using GenomeStudio (Illumina Inc.), colour-corrected [48], and batch-normalised. Correction for probe type using SWAN [49] and removal of probes targeting known SNPs was also performed [50]. Output data were reported as β values, representing DNA methylation at each locus within a range of 0–1. Each candidate gene was associated with a range of 3 to 22 CpG probes, except *NR3C1* for which 37 associated CpG probes were located on the array. These represented both upstream (enhancers and promoters) and gene body regions. Significant differences in candidate gene DNA methylation between groups were identified by any associated CpG exhibiting a *P*-value of <0.05, using a Mann-Whitney test. Since the methylation detection limit for a single CpG by bisulfite pyrosequencing is estimated to be ∼5% [51], candidate CpG sites for further assessment using this method were selected only when the difference in β values (Δβ) were ≥7% between controls and cases. Additionally, in situations where >1 CpG site per gene fit this criteria, the site with the largest Δβ was selected; however, the genomic location with respect to promoter or CpG island proximity was also considered. In the case of *NR3C1* two Illumina CpG sites were selected for further analysis, to allow for a more comprehensive assessment of the gene region proximal to promoter 1D. In the case of *HSD11B2*, 11 CpG sites were interrogated within the promoter region and included sites that were shown to be differentially methylated in IUGR in the literature [52].

### Bisulfite Pyrosequencing

To assess CpG methylation in our extended cohort of controls and cases, placental DNA samples were analysed using bisulfite pyrosequencing. Forward, reverse and sequencing primers for bisulfite converted DNA were designed with the aid of Pyro Q-CpG software (Qiagen Ltd., GmbH, Hilden, Germany; Table S1). PCR reactions consisted of ∼20 ng bisulfite-converted DNA, 1× PCR buffer (Qiagen Ltd.), 0.2 mM dNTPs (Invitrogen, Carlsbad, CA), 0.4 µM forward and reverse primers (Integrated DNA Technologies Inc., Coralville, IA) and 0.18 U DNA polymerase (HotStarTaq, Qiagen Ltd.). Optimised PCR conditions consisted of 95°C for 15 min, 40 cycles of 95°C for 30 sec, variable annealing temperature (Ta) for 30 sec, 72°C for 30 sec, and finally 72°C for 10 min. The optimal Ta was 51°C for *CYP19*, 55°C for *NR3C1* assay 1 and 2, *CRHBP*, *CYP11A1*, *HSD3B1*, *HSD11B2* and *TEAD3,* and 57°C for *CRH* PCR reactions. PCR products were sequenced and DNA methylation analysed at each CpG site using the Pyromark Q96 MD Pyrosequencer (Qiagen Ltd.). Samples from each patient group were equally allocated to multiple pyrosequencing plates to cancel out biases due to inter-plate variability (<5% for each assay). Where possible, more than one CpG site was included in the pyrosequencing assay, in addition to the site identified using the DNA methylation array.

### Gene Expression Array

Gene expression data were obtained using a subset of gestational age and gender matched control (n = 8) and EOPET (n = 8) cases that were run on the Illumina DNA methylation array. RNA was extracted from one chorionic villus site stored at −80°C in RNAlater (Invitrogen), using the RNeasy kit (Qiagen Ltd.) following the manufacturer’s protocol. RNA was assessed for quality using the Bioanalyzer 2100 (Agilent, Santa Clara, CA), reverse transcribed to cDNA (TotalPrep RNA Amplification kit, Illumina, Inc.) and hybridised to a HT-12v4 Expression BeadChip (Illumina, Inc.) according to the manufacturer’s protocol. This chip interrogates >47,000 transcripts genome-wide. Raw data were quantile normalised using GenomeStudio 2011 (Illumina, Inc.) and probes with bad detection *P*-values were removed and replaced with a value of zero. The full data set is being published separately (Blair *et al*., in preparation; GEO: GSE44711). Expression data were available for all genes followed up in this study.

### NR3C1 Isoform-specific Polymerase Chain Reaction (PCR)

*NR3C1* exon 1C and exon 1D isoform-specific PCR was performed across five chorionic villus samples obtained over different gestational time points, including 7, 19, 24, 32 and 40 weeks of gestation. RNA was extracted from tissue samples stored at −80°C in RNAlater using the phenol-chloroform method (TRIzol, Invitrogen) according to the manufacturer’s protocol. Commercially available adult male hippocampus RNA was used as a positive control for *NR3C1* exon 1D expression (BioChain Institute, Inc., Newark, CA). RNA concentrations and purities were measured using the Nanodrop 1000 spectrophotometer (ThermoScientific, Wilmington, DE). cDNA was prepared from 200 ng RNA using the High Capacity RT kit (Applied Biosystems Inc., Foster City, CA). A negative control reaction was prepared through the omission of reverse transcriptase. PCR primer sequences were exon-exon spanning: *NR3C1* exon 1D forward primer 5′-ACCCTAAACCCACACAGCAC-3′, *NR3C1* exon 1C forward primer 5′-GGGCAATGGGAGACTTTCTT-3′, and a shared *NR3C1* exon 1C/D reverse primer 5′-TCCATCACATCTCCCCTCTC-3′. Amplicon length for *NR3C1* exon 1C and 1D transcripts were 288 and 159 base pairs, respectively. PCR reactions were prepared as described in *section 2.4*, using 1 uL cDNA and a Ta of 60°C for both assays. PCR products were run on a 1% standard agarose gel at 120 V for 45 min, alongside a 100 base pair DNA ladder (Invitrogen) and visualised using a Universal Hood II gel imager and Quantity One v4.6.9 software (Bio-Rad, Hercules, CA).

### Statistics

Patient clinical data were compared using a Student’s t-test, non-parametric Mann-Whitney test or a chi-squared test where appropriate. DNA methylation array data for control and EOPET cases were compared using a Mann-Whitney test. For bisulfite pyrosequencing analyses, Spearman’s Rho correlation coefficients were calculated to determine CpG correlation within an assay. Univariate linear regression analyses were performed in control and case cohorts to identify potential confounding factors that influence mean DNA methylation at a selected gene. Variables assessed included birth weight, gestational age at birth, maternal age, gender of infant and the presence/absence of labour. If confounding factors were absent, an analysis of variance was used to compare control, EOPET, LOPET and nIUGR groups. If confounding factors were present, an analysis of covariance correcting for that variable was applied. All analyses included a Levene’s test for equality of error variances and were followed by Bonferroni post-hoc tests for multiple comparisons. RNA expression array data for control and EOPET cases were compared using a Student’s t-test or Mann-Whitney test as appropriate. *P*-values of <0.05 were considered significant.

## Results

### Candidate Genes for Differential Placental DNA Methylation in Pre-eclampsia

**Figure 1:**
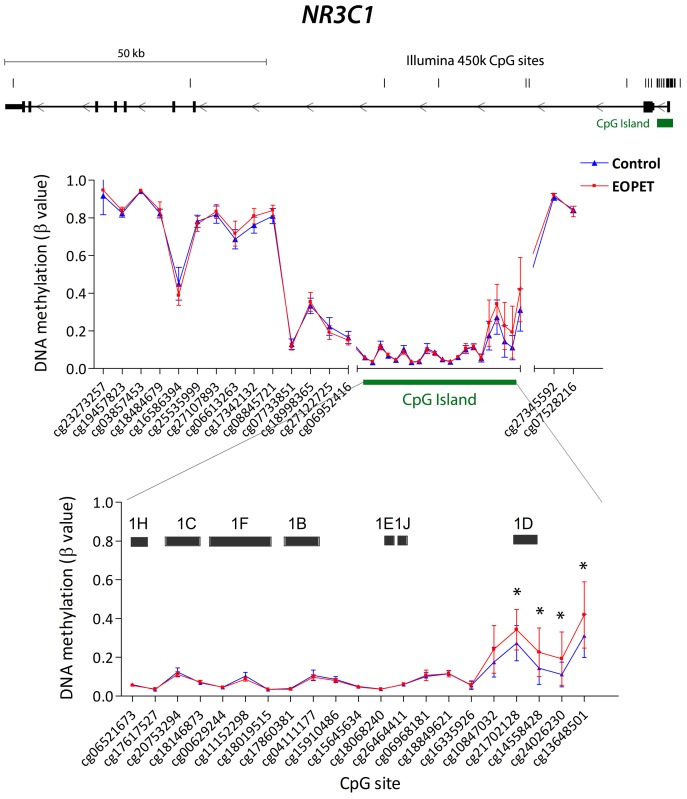
CpG sites on the Illumina Infinium HumanMethylation450 BeadChip array across the *NR3C1* gene region. Upper graph: DNA methylation at each CpG site in control (n = 19) and early onset pre-eclampsia (EOPET; n = 19) placentae. Lower graph: enlargement of region associated with CpG island containing multiple alternative first exons (black boxes). The Illumina CpG probe identifier is indicated by cg# and the position of CpG sites on the graphs are not to scale. *P<0.05.

Multiple genes associated with stress pathways and steroid production were associated with differentially methylated CpG sites in EOPET cases compared with controls based on the Illumina DNA methylation array (Table 2). In the region containing alternative *NR3C1* exon 1s and respective upstream promoters, differential DNA methylation was observed at CpG sites adjacent and upstream of exon 1D (Fig. 1). In EOPET placentae there was significant hypermethylation of these sites compared to controls (Fig. 1; Table 2). Interestingly, no other *NR3C1* promoter regions, including the widely studied exon 1F region, were altered in EOPET placentae. In fact, in normal placentae CpG sites across the *NR3C1* promoter region were largely unmethylated (β value 0–0.1), with the exception of exon 1D in which DNA methylation values were greater (β value 0.1–0.3; Fig. 1).

**Table 2:** Illumina Infinium HumanMethylation450 BeadChip array data.

Gene	Chromosome location	CpG of interest	Gene region	CpG island?	DNA methylation (β value)		Δ β	*P*-value
					Control	EOPET		
*NR3C1*	ch5 (q31.3)	cg14558428	exon 1D promoter	Y	0.14±0.08	0.23±0.12	0.09	0.01
*NR3C1*	ch5 (q31.3)	cg24026230	exon 1D promoter	Y	0.11±0.06	0.19±0.13	0.08	0.02
*CRH*	ch8 (q13.1)	cg23409074	exon 1 (5′ UTR)	N	0.37±0.12	0.29±0.06	−0.08	0.03
*CRHBP*	ch5 (q13.3)	cg13777717	intron 3	Y	0.40±0.08	0.48±0.07	0.08	0.001
*CYP11A1*	ch15 (q24.1)	cg06285340	exon 1	N	0.54±0.03	0.42±0.04	−0.12	<0.0001
*HSD3B1*	ch1 (p12)	cg16175792	exon 2	N	0.82±0.02	0.69±0.05	−0.13	<0.0001
*TEAD3*	ch6 (p21.31)	cg10893014	intron 1	N	0.60±0.06	0.39±0.10	−0.21	<0.0001
*CYP19*	ch15 (p21.2)	cg15329467	placental-specific exon 1.1 promoter	N	0.56±0.05	0.46±0.05	−0.10	<0.0001

The Illumina CpG of interest, gene location and CpG island (defined as length >200 base pairs, 50% GC content and >0.6 CG ratio) association is provided. Mean ± SD methylation β values and *P*-values are given for each gene for control (n = 19) and early onset pre-eclampsia (EOPET; n = 19) placental samples. *NR3C1*: nuclear receptor subfamily 3, group C, member 1, *CRHBP*: corticotropin releasing hormone (CRH) binding protein: *HSD3B1*∶3β-hydroxy-delta-5-steroid dehydrogenase type 1, *TEAD3*: TEA domain family member 3.

Regions associated with other candidate genes also exhibited altered placental DNA methylation patterns in control versus EOPET cases (Table 2). A CpG island associated with *CRHBP* (Fig. S1) was significantly hypermethylated in EOPET, whereas non-CpG island regions associated with *CRH*, *CYP11A1*, *HSD3B1*, *TEAD3* and *CYP19* were hypomethylated in EOPET versus control placentae (Fig. S2-6, respectively). A CpG associated with the transcription factor *TEAD3* was particularly altered, with an average β value decrease of 0.21 in EOPET (*P*<0.0001). DNA methylation values between control and EOPET groups were altered by ≥0.1, or 10% points, at CpG sites within steroidogenic pathway genes including *CYP11A1*, *HSD3B1* and *CYP19* (all *P*<0.0001). Candidate genes that were assessed but revealed no alteration in placental DNA methylation patterns at individual CpG sites in control and EOPET cases included *CRHR1*, *HSD11B2*, *POMC*, *STARD3* and *CYP17* (data not shown).

Bisulfite pyrosequencing was performed to confirm DNA methylation differences at significant CpG sites in a larger cohort of controls as well as cases of LOPET and nIUGR. For control and EOPET samples that were assessed by both array and pyrosequencing techniques, DNA methylation values at investigated CpG sites for the two methods were highly correlated (all loci Spearman’s Rho>0.8; *P*<0.001). Within pyrosequencing assays, DNA methylation values at individual CpG sites were also correlated (Fig. S7), and therefore mean methylation values for each region were compared.

### Gestational Age, but not Presence/absence of Labour, is a Confounder of Placental DNA Methylation at Candidate Genes

Using the bisulfite pyrosequencing data, univariate linear regression analyses for each studied region were performed on control and case cohorts to determine whether specific known patient variables might influence DNA methylation in that cohort. Birth weight, maternal age and infant gender were not significant confounders in any group assessed (data not shown). Gestational age significantly influenced DNA methylation in third trimester control placentae (n = 111; range 28–40 weeks) in a gene dependent manner. Increasing gestational age was negatively associated with CpG methylation within targeted regions of *CRH* (R = −0.19; *P*<0.05), *CYP19* (R = −0.27; *P*<0.01), *TEAD3* (R = −0.51; *P*<0.0001) and *HSD3B1* (R = −0.36; *P*<0.001) genes, and positively associated with CpG methylation of *CRHBP* (R = 0.26; *P*<0.01). However, there was no significant effect of gestational age on CpG methylation of the *NR3C1* exon 1D promoter region (R = −0.03) nor *CYP11A1* (R = −0.13). Consequently, gestational age was corrected for in those genes in which it was found to be a confounder. It should be noted that even though the EOPET cases were on average of an earlier gestational age than controls, the methylation trends in EOPET were generally in the opposite direction than expected based on these gestational age associations.

Since the process of labour can induce gene expression changes in the placenta [53], [54], we assessed whether or not labour affected DNA methylation at the genes included in this study. In a clearly defined subset of control patients, those that experienced labour (n = 46) and those that did not (n = 8), were compared. Although sample size in the non-labour group was small and mode of delivery data were not available for the full cohort, preliminary data suggested no significant DNA methylation changes at any of the genes/CpG sites studied as a result of labour (data not shown). As changes in DNA methylation may be dependent on cell replication, labour would not be expected to be associated with large changes in DNA methylation, and this confirms that the methylation changes we observed are unlikely to be altered by short term exposures at the end of pregnancy.

### Glucocorticoid Signalling Pathway Genes are Differentially Methylated in Pre-eclampsia

**Figure 2:**
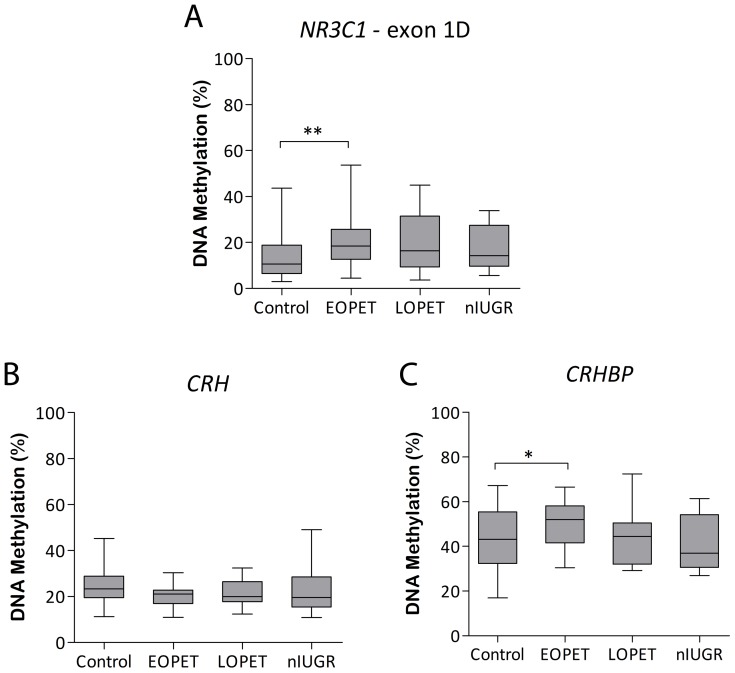
Placental DNA methylation of genes involved in cortisol signalling and bioavailability. DNA methylation at CpG sites within A) exon 1D promoter of the nuclear receptor subfamily 3, group C, member 1 (*NR3C1*), B) corticotropin releasing hormone (*CRH*) and C) CRH binding protein (*CRHBP*) genes. Control, early onset pre-eclampsia (EOPET), late onset pre-eclampsia (LOPET) and normotensive intrauterine growth restriction (nIUGR) placentae are compared. Median and interquartile ranges are given based on average assay CpG methylation measured by bisulfite pyrosequencing. *P<0.05, **P<0.01.

Differential placental DNA methylation of *NR3C1*, proximal to the exon 1D promoter, was verified in EOPET cases at multiple CpG sites by bisulfite pyrosequencing. Average DNA methylation values were significantly increased across the *NR3C1* exon 1D region compared with controls (+8.46%; *P*<0.01; Fig. 2A). In addition, there were trends indicative of increased DNA methylation at this locus in LOPET placentae relative to controls (+6.4%; *P* = 0.09; Fig. 2A). Increased promoter methylation of *NR3C1* could be a feature of both early and late pre-eclampsia, but perturbations are more pronounced in the earlier onset cases. Alternatively, a subset of cases classified as LOPET may have a placental aetiology more similar to EOPET, causing a trend towards similar changes. While *NR3C1* DNA methylation trends for nIUGR cases appeared similar to that of LOPET placentae, mean methylation differed from controls by <5% and was not significantly altered (Fig. 2A).

Genes involved in the hormonal regulation of glucocorticoid levels were also followed up with bisulfite pyrosequencing. In line with DNA methylation array findings, there was a non-significant trend for decreased CpG methylation at the *CRH* gene in EOPET placentae (5′ UTR; −4.30%; *P* = 0.11; Fig. 2B) and an opposing significant increase in DNA methylation associated with *CRHBP* (intron 3; +9.14%; *P*<0.05; Fig. 2C), compared with controls. However, DNA methylation at these CpG sites was not significantly altered in LOPET or nIUGR placentae relative to controls.

### Steroidogenic Pathway Genes are Differentially Methylated in Pre-eclampsia

**Figure 3:**
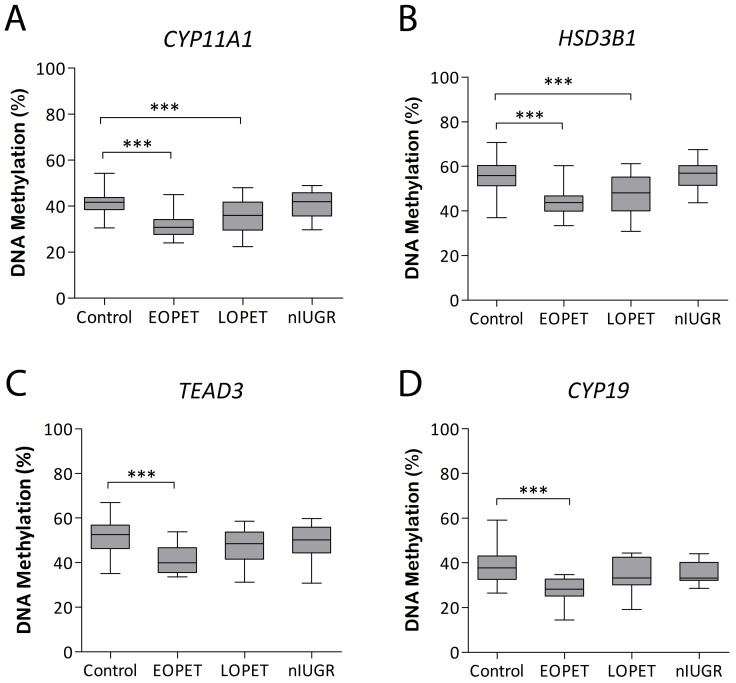
Placental DNA methylation of genes involved in steroidogenesis. DNA methylation at CpG sites within A) *CYP11A1*, B) 3β-hydroxl-delta-5-steroid dehydrogenase type I (*HSD3B1*), C) TEA domain family member 3 (*TEAD3*) and D) *CYP19* genes. Control, early onset pre-eclampsia (EOPET), late onset pre-eclampsia (LOPET) and normotensive intrauterine growth restriction (nIUGR) placentae are compared. Median and interquartile ranges are given based on average assay CpG methylation measured by bisulfite pyrosequencing. ***P<0.001.

The shift in DNA methylation at Illumina CpG sites associated with steroidogenic genes was confirmed in pre-eclampsia-associated placentae by bisulfite pyrosequencing. In EOPET there were significant losses of DNA methylation at regions associated with *CYP11A1* (exon 1; −9.66%; *P*<0.00001; Fig. 3A) and *HSD3B1* (exon 2; −12.49%; *P*<0.00001; Fig. 3B) compared with controls. In addition, the same loci were perturbed in LOPET cases (*CYP11A1*: –.5.77%; *P*<0.001 and *HSD3B1*: −6.88%; *P*<0.001). *TEAD3* was significantly hypomethylated in EOPET compared with controls (intron 1; −12.56%; *P*<0.00001), but not in LOPET placentae (Fig. 3C). Similarly, hypomethylation of CpG sites associated with *CYP19* in EOPET cases (exon 1.1; −10.6%; *P*<0.0001; Fig. 3D), was not present in LOPET cases compared with control placentae. In addition, there was no significant association of isolated nIUGR with placental DNA methylation found for any of the steroidogenic genes studied (Fig. 3).

### DNA Methylation of the HSD11B2 Promoter is not Altered in nIUGR

**Figure 4:**
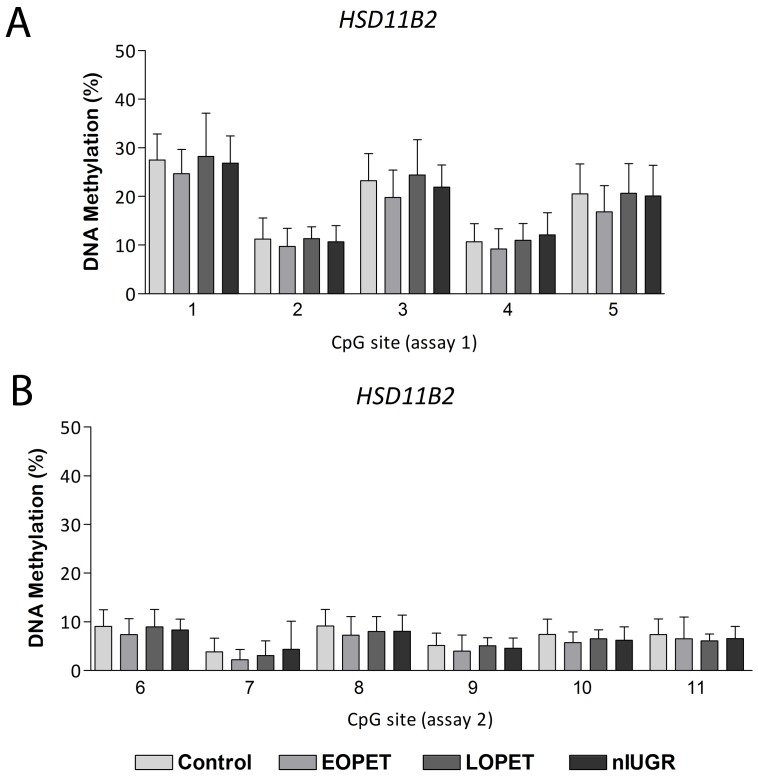
Placental DNA methylation of the 11β-hydroxysteroid dehydrogenase type 2 (*HSD11B2*) promoter. Control, early onset pre-eclampsia (EOPET), late onset pre-eclampsia (LOPET) and normotensive intrauterine growth restriction (nIUGR) placentae are compared. Mean ± SD DNA methylation values are given for consecutive CpG sites in *HSD11B2* assay 1 (A) and assay 2 (B) measured by bisulfite pyrosequencing.

We elected to assess *HSD11B2* promoter DNA methylation by bisulfite pyrosequencing in our nIUGR cohort, targeting 11 consecutive CpG sites including four CpG sites previously associated with birth weight across normal ranges and increased DNA methylation in IUGR cases [52]. Methylation values at different CpG sites across this region were variable and were not correlated (Fig. S7); therefore DNA methylation was assessed at individual CpG sites. Univariate linear regression analyses revealed no confounding effect of gestational age, maternal age, infant gender or mode of delivery (data not shown). Also, there was no significant relationship between birth weight and *HSD11B2* promoter DNA methylation across normal birth weight ranges in our cohort of 111 controls (Table S2). Furthermore, we did not observe altered *HSD11B2* promoter methylation between control and nIUGR cases (Fig. 4A and B). Pyrosequencing for EOPET and LOPET cohorts also confirmed no change in DNA methylation values at this locus in pre-eclampsia (Fig. 4A and B), as previously indicated by DNA methylation array data.

### DNA Methylation is Inversely Associated with mRNA Expression for Selected Genes

Gene expression array data were available for a small subset of matched control and EOPET cases overlapping the larger cohort. Significant upregulation of *CRH* (>20 fold), *CYP11A1* (4 fold), *HSD3B1* (5 fold) and *CYP19* (5 fold) transcripts was observed in EOPET cases compared to controls (Table 3; all *P*<0.01) but not *TEAD3*. Also, there were trends for the downregulation of *CRHBP* and upregulation of *HSD11B2* expression in EOPET-associated placentae (Table 3). The expression of *NR3C1* mRNA was not altered; however, as the probe to detect *NR3C1* mRNA was not transcript-specific, it is possible that subtle changes in isolated transcript expression would not be detected.

**Table 3:** Illumina HT-12v4 Expression BeadChip gene expression array data for genes followed up by bisulfite pyrosequencing.

Gene	Array probe	RNA expression (Fluorescent Units)		*P*-value
		Control	EOPET	
*NR3C1*	ILMN_2389347	59.4±16.3	57.8±12.2	NS
*CRH*	ILMN_1668035	64.22±20.6	1431±1124	<0.01
*CRHBP*	ILMN_1761312	153.9±137.6	90.7±90.5	NS
*CYP11A1*	ILMN_1768820	442.8±277.2	1750±1377	<0.01
*HSD3B1*	ILMN_1780693	238.4±120.2	1193±857	<0.01
*TEAD3*	ILMN_1814002	118.9±44.9	119.9±38.8	NS
*CYP19*	ILMN_2387860	112.0±54.6	671.4±579.8	<0.01
*HSD11B2*	ILMN_1813350	37.4±21.5	148.6±294.2	NS

The Illumina probe identifier, mean ± SD expression values and the associated *P*-value is given for each gene for a subset of control (n = 8) and early onset pre-eclampsia (EOPET; n = 8) placental samples. *NR3C1*: nuclear receptor subfamily 3, group C, member 1, *CRHBP*: corticotropin releasing hormone (CRH) binding protein: *HSD3B1*∶3β-hydroxy-delta-5-steroid dehydrogenase type 1, *TEAD3*: TEA domain family member 3, *HSD11B2*∶11β-hydroxysteroid dehydrogenase type 2.

In addition, gene expression across both control and EOPET groups was inversely correlated with DNA methylation in promoter regions of *CRHBP* (R = −0.70, *P*<0.01), *CYP11A1* (R = −0.76; *P*<0.01), *HSD3B1* (R = −0.55; *P* = 0.05) and *CYP19* (R = −0.36) genes, although not *CRH* (R = −0.05).

### The NR3C1 Exon 1D Transcript is Expressed in Placenta Across Gestation

**Figure 5:**
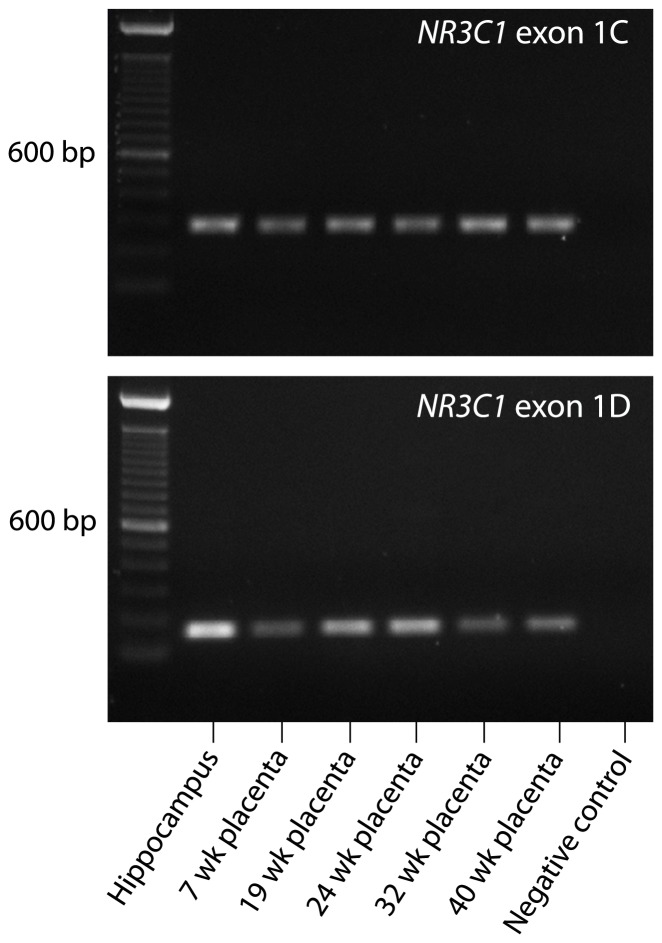
*NR3C1* isoform expression in the human placenta. Expression of *NR3C1* exon 1C and 1D alternative transcripts in the placenta across gestation. A cDNA reaction prepared from adult male hippocampus RNA was used as a positive control. The negative control consisted of a cDNA reaction omitting reverse transcriptase. *NR3C1* exon 1C and 1D PCR amplicons were 288 base pairs (bp) and 159 bp in length, respectively.

Isoform-specific PCR was performed for *NR3C1* exon 1C and exon 1D to determine their expression in the placenta over gestation (Fig. 5). The ubiquitously expressed exon 1C was observed across all gestational ages assessed including 7, 19, 24, 32 and 40 week gestationally aged placental samples. The exon 1D transcript was also expressed in the placenta across these developmental stages. Since this transcript has been previously reported only in the adult hippocampus, and absent from a range of other tissues [55], expression of *NR3C1* exon 1D may be of particular importance in the placenta.

## Discussion

The majority of normotensive pregnancies are characterised by the absence of placental cortisol; however, cortisol is detectable in the placenta of up to 80% of pre-eclampsia-associated pregnancies [24]. This is of concern given the overwhelming evidence for the role of glucocorticoids in the developmental programming of disease. This is documented in both animal and human studies, and is associated with low birth weight, hypersensitive stress response and neurobehavioural anomalies in infants, and increased risk of metabolic, cardiovascular and reproductive syndromes in adults (reviewed in [1], [56], [57]). Determining the types of pregnancies that are at elevated risk for such programming events and understanding the mechanisms that lead to these changes may permit future advancements in obstetrical and neonatal care and improved long-term health outcomes.

The human *NR3C1* gene contains multiple tissue-specific alternative first exons, each containing upstream promoters that can be silenced by DNA methylation [55], [58]. Previously, altered DNA methylation at specific CpG sites within the exon 1_7_ promoter in rat hippocampi [31], [59], and in the human orthologue exon 1F in brain, peripheral and cord blood, and placenta [32], [34], [35], [37], has been associated with perinatal or prenatal stress. In the placenta, DNA methylation at *NR3C1* exon 1F promoter is associated with neurological and mental health outcomes in infants in normal pregnancy [36] and in babies large for gestational age [60]. The biological impact of the small DNA methylation changes (1–5% points) reported at the exon 1F promoter, a region exhibiting very low levels of methylation (<10%), is not clear.

In this study, promoter-wide DNA methylation profiling revealed differential patterns at the exon 1D promoter, but not at exon 1F. Levels of DNA methylation in the exon 1D region ranged from 5–40% in normal placenta, agreeing with previous reports of large interindividual variation within the *NR3C1* promoter [61]. While this interindividual variation was also present in pathological placentae, DNA methylation values were on average significantly higher than normal placentae, providing evidence that at least in the human placenta, exon 1D may be more susceptible to environmental insults than alternative promoter regions. The interrogated CpG sites were proximal to the putative consensus sequence (CGCCATnTT) for the ubiquitously expressed zinc finger transcription factor Yin Yang 1 (YY1), in addition to various other transcription factor motifs located in the exon 1D promoter [61]. Given the proximity of these consensus sequences to regions of differential DNA methylation in our study, we speculate that altered DNA methylation may be representative of an altered transcriptional landscape in these cases. In addition to gene expression being transiently influenced by events in the perinatal period, such as mode of delivery, it can also be affected by placental processing time; while such factors have not been shown to have major effects on DNA methylation [62].

Given the complexity of *NR3C1* gene regulation and the fact that alternative splice variants are expressed in the placenta before and after labour [54], we wished to determine if the differentially methylated promoter, exon 1D, was expressed in the placenta. Previous reports indicated that exon 1D was exclusively expressed in adult hippocampal tissue and was absent from a panel of adult peripheral tissues and cells [55]. We found that *NR3C1* exon 1D is expressed in the human placenta over gestational ages ranging from the first to the third trimester. Of interest, this promoter region exclusively maps to histone-3 lysine-4 mono-methylation ‘active’ marks across numerous cell lines, is predicted to be within a strong enhancer region according to ENCODE and is contained within a DNase I hypersensitivity cluster. These observations suggest a regulatory role for the promoter region upstream of exon 1D that could be involved in overall *NR3C1* expression.

During development the HPA axis is susceptible to altered programming, of which the CRH signalling system is a target [21]. In rats, *in utero* exposure to stress elevates cortisol in offspring, which is associated with increased hypothalamic *CRH* expression [63], and decreased CRHBP and increased ACTH plasma concentrations [64], [65], indicating increased adrenal cortisol production and bioavailability. Like the hypothalamic isoform, placental CRH can stimulate maternal and fetal adrenal steroidogenesis, as well as placental hormone production, specifically repressing progesterone [66] and augmenting estrogen [14] synthesis in trophoblasts.

In women with high third trimester choline supplementation, a 4–5% increase in DNA methylation at CpG sites within the *CRH* promoter in placenta was associated with a ∼33% fall in placental *CRH* transcript levels and cord blood cortisol concentration, suggesting a direct effect on the fetal HPA axis [41]. As these DNA methylation changes were observed at CpG sites adjacent to the sites included in our study, an equivalent decrease in DNA methylation levels in EOPET compared to controls may reflect increased transcript levels. Indeed, placental CRH content and umbilical cord and maternal plasma CRH are significantly elevated in pre-eclampsia-associated pregnancies compared to normal pregnancies [67]–[71]. In this study, we further confirmed an increase in placental *CRH* mRNA in a subset of pre-eclampsia cases, although we did not observe a relationship between DNA methylation at these particular CpG sites and transcript expression in this small number of cases.

Elevated CRH has been reported in umbilical cord plasma of IUGR infants [72]. However, we found no change in *CRH* DNA methylation levels in placentae of nIUGR pregnancies in our study. While multiple regulatory processes in addition to DNA methylation can influence *CRH* gene expression, it is also possible that IUGR cases utilised in other studies included cases with co-existing hypertension/pre-eclampsia that might have contributed to altered CRH levels. Increased *CRHR1* gene expression was also reported in placentae of complicated pregnancies including pre-eclampsia and IUGR [73]; however, we did not detect altered DNA methylation of the *CRHR1* gene in this study.

Intragenic DNA methylation can be associated with altered gene expression, and may regulate alternative promoters [74]. In EOPET-associated placentae, we observed hypermethylation of *CRHBP* at CpG sites contained within a CpG island overlapping the third intron. There was an inverse relationship between DNA methylation at this region and RNA expression across a subset of control and EOPET cases and a trend for downregulation of *CRHBP* mRNA in pre-eclampsia-associated placentae. Other studies have found reciprocal decreases in *CRHBP* and increases in *CRH* transcripts in the placenta [75] or protein levels in the plasma [70] of women who developed pre-eclampsia. Thus, elevated levels of circulating CRH, through increased placental CRH and/or decreased CRHBP production may be a mechanism to stimulate increased downstream glucocorticoid synthesis.

The physiological elevation of maternal glucocorticoids during the third trimester is normally counteracted by the concurrent upregulation of placental *HSD11B2* [25]. Adverse effects of glucocorticoid exposure through the inhibition of placental *HSD11B2* expression/11βHSD type 2 activity in offspring are well documented [19], [20], [22], [76], and have been associated with hypermethylation of CpG sites within the *Hsd11b2* promoter in rats [40]. Despite lowered placental *HSD11B2* expression being reported in pre-eclampsia [23], [24], DNA methylation patterns across the *HSD11B2* promoter and gene regions were not altered in our cohort. Additionally, we found only a moderate trend for *HSD11B2* mRNA downregulation in our sample subset. Furthermore, there was no relationship between *HSD11B2* promoter DNA methylation and birth weight, as indicated in a previous study [52].

To sustain considerable estrogen and progesterone synthesis required for placental function and pregnancy maintenance, the placental syncytiotrophoblast abundantly express steroidogenic pathway genes [77]. In the placenta, a structural homologue to steroidal acute regulatory protein, *STARD3*, transports cholesterol for conversion to pregnenolone by cytochrome P450 side chain cleavage (encoded by *CYP11A1*) [78], [79]. DNA methylation of CpG sites at the promoters of *STARD3* and *CYP11A1* genes was assessed given their rate limiting involvement in placental steroidogenesis. Placental DNA methylation patterns associated with *STARD3* were unaltered among cases; however, there was significant hypomethylation of *CYP11A1* in pre-eclampsia cases compared with controls. Furthermore, our findings of upregulation of *CYP11A1* in pre-eclampsia-associated placentae, and a negative correlation between placental *CYP11A1* DNA methylation and RNA expression support a role for DNA methylation in regulating *CYP11A1* expression. The transcription factor activating protein 2 (AP2) is proposed to transactivate *CYP11A1* in the placenta [80] and the binding of this factor to gene promoters is sensitive to DNA methylation in other tissues [81]. Based on the DNA methylation array data, the differential DNA methylation observed in pre-eclampsia-associated placentae is likely to extend into this consensus sequence located ∼100 base pairs upstream of interrogated CpG sites.

The placental production of up to 250–300 mg progesterone per day is maintained by the conversion of pregnenolone into progesterone by 3β-hydroxysteroid dehydrogenase type 1 (3βHSD1 encoded by *HSD3B1*) [77]. Placental-specific enhancers are located 2500–3000 base pairs upstream of the *HSD3B1* promoter, and contain a binding site for transcription enhancer factor 5 (TEF-5; encoded by *TEAD3*), which is predominantly expressed by the placenta [82] and necessary to confer placental-specific *HSD3B1* expression [83]. Regions associated with both *HSD3B1* (exon 2) and *TEAD3* (intron 1) were significantly hypomethylated in EOPET placentae suggesting a propensity for altered expression of steroidogenic genes. *HSD3B1* mRNA was significantly elevated in EOPET placentae. Therefore, loss of DNA methylation at exon 2 may be associated with increased 3βHSD1 levels in the placenta and enhanced production of steroid hormone precursors. We did not find altered expression of *TEAD3* mRNA in EOPET cases; however, mRNA expression in the third trimester may not represent transcript expression across gestation. Since DNA methylation is relatively stable, it is possible that these differences reflect expression earlier in gestation, and that the differential methylation levels represent an altered chromatin structure that permits interaction with transcription factors at specific gestational time points.

Steroidogenic genes necessary for estrogen synthesis, including *CYP17* (17α-hydroxylase; synthesises androgenic precursors) and *CYP19* (aromatase) were also studied. While DNA methylation of *CYP17* was not altered, significant hypomethylation was observed in the *CYP19* promoter in EOPET placentae. Human *CYP19* expression is controlled by tissue-specific promoters upstream of alternative first exons [84]. In this study, the differentially methylated CpG region in EOPET was contained within the placental-specific *CYP19* exon 1.1 promoter [85]. At other tissue-specific human *CYP19* promoters, DNA methylation has been associated with *CYP19* expression [86], [87]. We found significant up-regulation of *CYP19* mRNA in EOPET placentae and a trend for a negative association between *CYP19* DNA methylation of the exon 1.1 promoter and transcript expression. Therefore, DNA methylation may also play a prominent role in tissue-specific regulation of placental *CYP19*; however a larger sample size is required to confirm this.

It was somewhat surprising that the altered DNA methylation found in cortisol-signalling and hormone production genes was limited to pre-eclampsia and not observed in nIUGR cases. Birth weight was not a predictor of DNA methylation in any of the genes/CpG sites included in this study. Pre-eclampsia was accompanied by IUGR in 14/19 EOPET and 5/18 LOPET cases; however, we did not find significant effects of IUGR within pre-eclampsia groups. It is important to include isolated IUGR cases as a control group to delineate the effects of fetal and placental growth restriction with that of pre-eclampsia. Furthermore, the majority of DNA methylation changes were limited to early onset rather than late onset disease, supporting the idea that EOPET is aetiologically distinct to LOPET and that the cut-off of 34 weeks can distinguish cases with relatively greater placental involvement, rather than manifestations of disease primarily in the mother [88]–[92]. However, there was some overlap, in which hypomethylation of *CYP11A1* and *HSD3B1* genes was present in LOPET cases. Certain changes may occur in response to common features such as exposure to anti-hypertensive medication, even if the underlying pathology is different. Changes unique to EOPET may reflect more fundamental aetiological differences.

**Figure 6:**
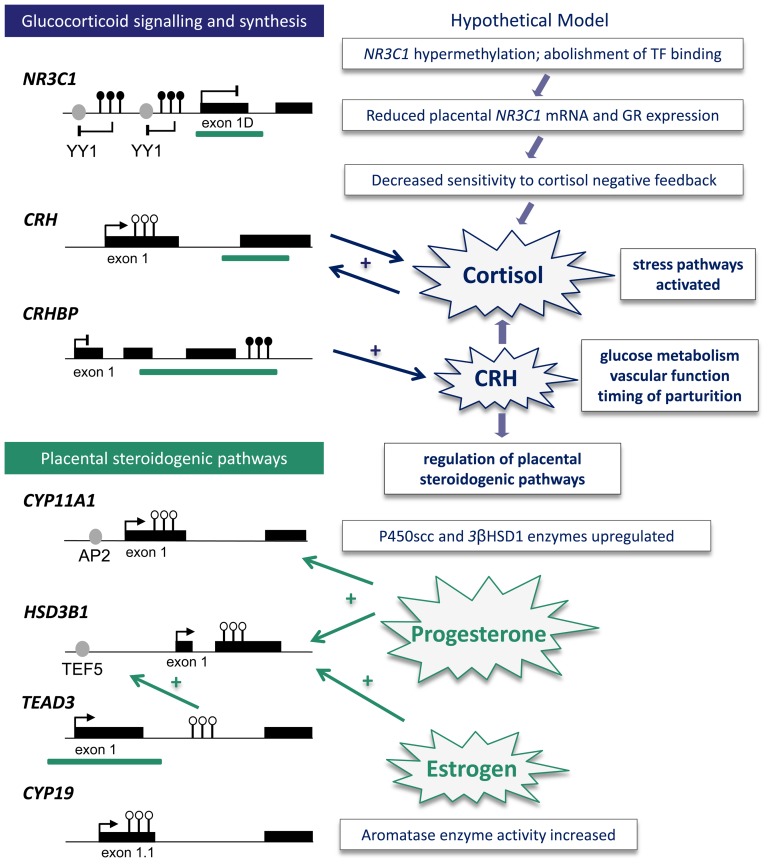
Model of altered stress and hormonal signalling gene-pathways in pre-eclampsia. Left hand side: summary of altered DNA methylation at specific CpG sites across candidate genes, where black and white circles indicate gain or loss of methylation, respectively. Coding regions are indicated by black rectangles and transcription factor (TF) binding motifs are represented by grey circles and include yin yang 1 (YY1; putative), activated protein 2 (AP2; known) and transcription enhancer factor 5 (TEF5; known). Bent lines represent transcription start sites: arrow or blunt heads hypothesise increased or decreased expression, respectively. Green bars represent CpG islands. Annotations are of approximate location and distance. Right hand side: hypothesised model of the downstream effects of altered placental DNA methylation at candidate genes. *NR3C1*: nuclear receptor subfamily 3, group C, member 1, *CRH*: corticotropin releasing hormone, *CRHBP*: CRH binding protein, *HSD3B1*∶3β-hydroxy-delta-5-steroid dehydrogenase type 1, *TEAD3*: TEA domain family member 3, GR: glucocorticoid receptor, *HSD11B2*∶11β-hydroxysteroid dehydrogenase type 2, P450scc: P450 side chain cleavage, 3βHSD1∶3β-hydroxysteroid dehydrogenase type 1.

We report altered DNA methylation proximal to genomic regions previously characterised as important for gene regulation, which is correlated with RNA expression in some cases. These data may represent common dysregulation of stress and hormonal pathways in pre-eclampsia. The biological consequences of altered expression of cortisol signalling and endocrine genes in the placenta in pre-eclampsia are likely to be complex and are hypothetically modelled in Figure 6. Reduced placental GR activation in combination with elevated CRH output may stimulate both maternal and fetal adrenal cortisol synthesis [10], [12] thus aberrantly increasing fetal exposure to glucocorticoids during development. Elevated CRH may also exert direct effects on placental function, including effects on glucose uptake and vascular tone and may contribute to the premature timing of labour common to pre-eclampsia [15]. While CRH stimulates placental estrogen synthesis (*CYP19* expression) [14], its repressive effect on progesterone synthesis (*CYP11A1* and *HSD3B1* expression) [66] may be dampened in the pre-eclampsia-associated placenta. Maternal progesterone, but not estrogen, concentrations are increased in women with preeclampsia [93], [94] and both steroid hormones positively stimulate *CYP11A1* and *HSD3B1* expression in trophoblast cells [95]. Therefore elevated progesterone, despite elevated CRH, may act in a compensatory feed-forward loop to promote placental steroidogenesis.

In summary, we suggest that changes in DNA methylation may contribute to the altered expression of cortisol-signalling genes previously linked to pre-eclampsia, and exclude this mechanism as an important regulatory factor in other related pregnancy complications. In addition, we highlight previously uncharacterised genes involved in placental steroidogenesis as differentially methylated in pre-eclampsia. Additional functional characterisation of these genes is required to further assess the relationship between DNA methylation and gene expression in the human placenta and will form the basis of future experiments. Unravelling which genetic pathways are susceptible to epigenetic modification in the placenta may provide a clearer aetiological perspective in disease cases and basis for targeted intervention.

## Supporting Information

Figure S1 **CpG sites on the Illumina Infinium HumanMethylation450 BeadChip array across the** ***CRHBP*** **gene region.** Upper panel: UCSC genome browser shot detailing location of Illumina CpG probes, histone marks predictive of enhancers, CpG island(s) and conserved transcription factor binding sites (TFBS) associated with the gene body. Lower panel: DNA methylation pattern at Illumina CpG sites across the corticotropin releasing hormone binding protein (*CRHBP*) gene region in control (n = 19) and early onset pre-eclampsia (EOPET; n = 19) placentae. The Illumina CpG probe identifier is indicated by cg# and the position of CpG sites on the graphs are not to scale. Values are mean ± SD, and *P<0.05 based on Mann-Whitney tests.
(DOCX)

Figure S2 **CpG sites on the Illumina Infinium HumanMethylation450 BeadChip array across the** ***CRH*** **gene region.** Upper panel: UCSC genome browser shot detailing location of Illumina CpG probes, histone marks predictive of enhancers, CpG island(s) and conserved transcription factor binding sites (TFBS) associated with the gene body. Lower panel: DNA methylation pattern at Illumina CpG sites across the corticotropin releasing hormone (*CRH*) gene region in control (n = 19) and early onset pre-eclampsia (EOPET; n = 19) placentae. The Illumina CpG probe identifier is indicated by cg# and the position of CpG sites on the graphs are not to scale. Values are mean ± SD, and *P<0.05 based on Mann-Whitney tests.
(DOCX)

Figure S3 **CpG sites on the Illumina Infinium HumanMethylation450 BeadChip array across the** ***CYP11A1*** **gene region.** Upper panel: UCSC genome browser shot detailing location of Illumina CpG probes, histone marks predictive of enhancers, CpG island(s) and conserved transcription factor binding sites (TFBS) associated with the gene body. Lower panel: DNA methylation pattern at Illumina CpG sites across the *CYP11A1* gene region in control (n = 19) and early onset pre-eclampsia (EOPET; n = 19) placentae. The Illumina CpG probe identifier is indicated by cg# and the position of CpG sites on the graphs are not to scale. Values are mean ± SD, and *P<0.05 based on Mann-Whitney tests.
(DOCX)

Figure S4 **CpG sites on the Illumina Infinium HumanMethylation450 BeadChip array across the** ***HSD3B1*** **gene region.** Upper panel: UCSC genome browser shot detailing location of Illumina CpG probes, histone marks predictive of enhancers, CpG island(s) and conserved transcription factor binding sites (TFBS) associated with the gene body. Lower panel: DNA methylation pattern at Illumina CpG sites across the 3β-hydroxy-delta-5-steroid dehydrogenase type 1 (*HSD3B1*) gene region in control (n = 19) and early onset pre-eclampsia (EOPET; n = 19) placentae. The Illumina CpG probe identifier is indicated by cg# and the position of CpG sites on the graphs are not to scale. Values are mean ± SD, and *P<0.05 based on Mann-Whitney tests.
(DOCX)

Figure S5 **CpG sites on the Illumina Infinium HumanMethylation450 BeadChip array across the** ***TEAD3*** **gene region.** Upper panel: UCSC genome browser shot detailing location of Illumina CpG probes, histone marks predictive of enhancers, CpG island(s) and conserved transcription factor binding sites (TFBS) associated with the gene body. Lower panel: DNA methylation pattern at Illumina CpG sites across the TEA domain family member 3 (*TEAD3*) gene region in control (n = 19) and early onset pre-eclampsia (EOPET; n = 19) placentae. The Illumina CpG probe identifier is indicated by cg# and the position of CpG sites on the graphs are not to scale. Values are mean ± SD, and *P<0.05 based on Mann-Whitney tests.
(DOCX)

Figure S6 **CpG sites on the Illumina Infinium HumanMethylation450 BeadChip array across the** ***CYP19*** **gene region.** Upper panel: UCSC genome browser shot detailing location of Illumina CpG probes, histone marks predictive of enhancers, CpG island(s) and conserved transcription factor binding sites (TFBS) associated with the gene body. Lower panel: DNA methylation pattern at Illumina CpG sites across the *CYP19* gene region in control (n = 19) and early onset pre-eclampsia (EOPET; n = 19) placentae. The Illumina CpG probe identifier is indicated by cg# and the position of CpG sites on the graphs are not to scale. Values are mean ± SD, and *P<0.05 based on Mann-Whitney tests.
(DOCX)

Figure S7 **Assessment of assay CpG correlations where >1 CpG site was interrogated.** A) nuclear receptor subfamily 3, group C, member 1 (*NR3C1*), B) corticotropin releasing hormone (*CRH*), C) CRH binding protein (*CRHBP*), D) *CYP19*, E) TEA domain family member 3 (*TEAD3*) and F) 11β-hydroxysteroid dehydrogenase type 2 (*HSD11B2*). Spearman Rho (R) values and corresponding *P*-values are given based on bisulfite pyrosequencing results.
(DOCX)

Table S1 **Primer sequences for bisulfite pyrosequencing assays.** The genomic location of the PCR and individual CpG target, are based on Genome Browser hg19 version. F: forward, R: reverse, S: sequence, Bi: biotin label. *NR3C1*: nuclear receptor subfamily 3, group C, member 1, *CRH*: corticotropin releasing hormone, *CRHBP*: CRH binding protein: *HSD3B1*∶3β-hydroxy-delta-5-steroid dehydrogenase type 1, *TEAD3*: TEA domain family member 3, *HSD11B1*∶11β-hydroxysteroid dehydrogenase type 2.
(DOCX)

Table S2 ***HSD11B2*** **promoter methylation and infant birth weight.** Univariate linear regression analyses for the relationship between birth weight (measured in standard deviation points relative to sex and age-matched normal birth weight ranges) and DNA methylation of CpG sites within the 11β-hydroxysteroid dehydrogenase type 2 (*HSD11B2*) promoter in placenta.
(DOCX)

## References

[1] Harris A, Seckl J (2011) Glucocorticoids, prenatal stress and the programming of disease. Horm Behav 59: 279–289. 20591431 10.1016/j.yhbeh.2010.06.007

[2] Khong TY, De Wolf F, Robertson WB, Brosens I (1986) Inadequate maternal vascular response to placentation in pregnancies complicated by pre-eclampsia and by small-for-gestational age infants. Br J Obstet Gynaecol 93: 1049–1059. 3790464 10.1111/j.1471-0528.1986.tb07830.x

[3] Vince GS, Starkey PM, Austgulen R, Kwiatkowski D, Redman CW (1995) Interleukin-6, tumour necrosis factor and soluble tumour necrosis factor receptors in women with pre-eclampsia. Br J Obstet Gynaecol 102: 20–25. 7833306 10.1111/j.1471-0528.1995.tb09020.x

[4] Roberts JM, Hubel CA (2004) Oxidative stress in preeclampsia. Am J Obstet Gynecol 190: 1177–1178. 15167813 10.1016/j.ajog.2004.04.001

[5] von Dadelszen P, Magee LA, Roberts JM (2003) Subclassification of preeclampsia. Hypertens Pregnancy 22: 143–148. 12908998 10.1081/PRG-120021060

[6] Lewis G (2003) Beyond the numbers: Reviewing maternal deaths and complications to make pregnancy safer. Br Med Bull 67: 27–37. 14711752 10.1093/bmb/ldg009

[7] Hollenberg SM, Weinberger C, Ong ES, Cerelli G, Oro A, et al (1985) Primary structure and expression of a functional human glucocorticoid receptor cDNA. Nature 318: 635–641. 2867473 10.1038/318635a0 PMC6165583

[8] Munck A, Guyre PM, Holbrook NJ (1984) Physiological functions of glucocorticoids in stress and their relation to pharmacological actions. Endocr Rev 5: 25–44. 6368214 10.1210/edrv-5-1-25

[9] Riley SC, Walton JC, Herlick JM, Challis JR (1991) The localization and distribution of corticotropin-releasing hormone in the human placenta and fetal membranes throughout gestation. J Clin Endocrinol Metab 72: 1001–1007. 2022703 10.1210/jcem-72-5-1001

[10] King BR, Smith R, Nicholson RC (2001) The regulation of human corticotrophin-releasing hormone gene expression in the placenta. Peptides 22: 1941–1947. 11754985 10.1016/s0196-9781(01)00486-7

[11] Goland RS, Jozak S, Conwell I (1994) Placental corticotropin-releasing hormone and the hypercortisolism of pregnancy. Am J Obstet Gynecol 171: 1287–1291. 7977536 10.1016/0002-9378(94)90149-x

[12] Sirianni R, Rehman KS, Carr BR, Parker CR, Jr, Rainey WE (2005) Corticotropin-releasing hormone directly stimulates cortisol and the cortisol biosynthetic pathway in human fetal adrenal cells. J Clin Endocrinol Metab 90: 279–285. 15494460 10.1210/jc.2004-0865

[13] Beshay VE, Carr BR, Rainey WE (2007) The human fetal adrenal gland, corticotropin-releasing hormone, and parturition. Semin Reprod Med 25: 14–20. 17205420 10.1055/s-2006-956772

[14] You X, Yang R, Tang X, Gao L, Ni X (2006) Corticotropin-releasing hormone stimulates estrogen biosynthesis in cultured human placental trophoblasts. Biol Reprod 74: 1067–1072. 16467490 10.1095/biolreprod.105.049361

[15] Gangestad SW, Caldwell Hooper AE, Eaton MA (2012) On the function of placental corticotropin-releasing hormone: A role in maternal-fetal conflicts over blood glucose concentrations. Biol Rev Camb Philos Soc 87: 856–873. 22564253 10.1111/j.1469-185X.2012.00226.x

[16] Robinson BG, Emanuel RL, Frim DM, Majzoub JA (1988) Glucocorticoid stimulates expression of corticotropin-releasing hormone gene in human placenta. Proc Natl Acad Sci U S A 85: 5244–5248. 2839838 10.1073/pnas.85.14.5244 PMC281726

[17] Jones SA, Brooks AN, Challis JR (1989) Steroids modulate corticotropin-releasing hormone production in human fetal membranes and placenta. J Clin Endocrinol Metab 68: 825–830. 2537843 10.1210/jcem-68-4-825

[18] Edwards CR, Benediktsson R, Lindsay RS, Seckl JR (1993) Dysfunction of placental glucocorticoid barrier: Link between fetal environment and adult hypertension? Lancet 341: 355–357. 8094124 10.1016/0140-6736(93)90148-a

[19] Lindsay RS, Lindsay RM, Edwards CR, Seckl JR (1996) Inhibition of 11-beta-hydroxysteroid dehydrogenase in pregnant rats and the programming of blood pressure in the offspring. Hypertension 27: 1200–1204. 8641724 10.1161/01.hyp.27.6.1200

[20] Lindsay RS, Lindsay RM, Waddell BJ, Seckl JR (1996) Prenatal glucocorticoid exposure leads to offspring hyperglycaemia in the rat: Studies with the 11 beta-hydroxysteroid dehydrogenase inhibitor carbenoxolone. Diabetologia 39: 1299–1305. 8932995 10.1007/s001250050573

[21] Welberg LA, Seckl JR (2001) Prenatal stress, glucocorticoids and the programming of the brain. J Neuroendocrinol 13: 113–128. 11168837 10.1046/j.1365-2826.2001.00601.x

[22] Holmes MC, Abrahamsen CT, French KL, Paterson JM, Mullins JJ, et al (2006) The mother or the fetus? 11beta-hydroxysteroid dehydrogenase type 2 null mice provide evidence for direct fetal programming of behavior by endogenous glucocorticoids. J Neurosci 26: 3840–3844. 16597738 10.1523/JNEUROSCI.4464-05.2006 PMC6445356

[23] Schoof E, Girstl M, Frobenius W, Kirschbaum M, Dorr HG, et al (2001) Decreased gene expression of 11beta-hydroxysteroid dehydrogenase type 2 and 15-hydroxyprostaglandin dehydrogenase in human placenta of patients with preeclampsia. J Clin Endocrinol Metab 86: 1313–1317. 11238526 10.1210/jcem.86.3.7311

[24] Aufdenblatten M, Baumann M, Raio L, Dick B, Frey BM, et al (2009) Prematurity is related to high placental cortisol in preeclampsia. Pediatr Res 65: 198–202. 19047954 10.1203/PDR.0b013e31818d6c24

[25] Shams M, Kilby MD, Somerset DA, Howie AJ, Gupta A, et al (1998) 11Beta-hydroxysteroid dehydrogenase type 2 in human pregnancy and reduced expression in intrauterine growth restriction. Hum Reprod 13: 799–804. 9619527 10.1093/humrep/13.4.799

[26] McTernan CL, Draper N, Nicholson H, Chalder SM, Driver P, et al (2001) Reduced placental 11beta-hydroxysteroid dehydrogenase type 2 mRNA levels in human pregnancies complicated by intrauterine growth restriction: An analysis of possible mechanisms. J Clin Endocrinol Metab 86: 4979–4983. 11600574 10.1210/jcem.86.10.7893

[27] Dy J, Guan H, Sampath-Kumar R, Richardson BS, Yang K (2008) Placental 11beta-hydroxysteroid dehydrogenase type 2 is reduced in pregnancies complicated with idiopathic intrauterine growth restriction: Evidence that this is associated with an attenuated ratio of cortisone to cortisol in the umbilical artery. Placenta 29: 193–200. 10.1016/j.placenta.2007.10.010 18061258

[28] Waterland RA, Michels KB (2007) Epigenetic epidemiology of the developmental origins hypothesis. Annu Rev Nutr 27: 363–388. 17465856 10.1146/annurev.nutr.27.061406.093705

[29] Hogg K, Price EM, Hanna CW, Robinson WP (2012) Prenatal and perinatal environmental influences on the human fetal and placental epigenome. Clin Pharmacol Ther 92: 716–726. 23047650 10.1038/clpt.2012.141

[30] Wyrwoll CS, Kerrigan D, Holmes MC, Seckl JR, Drake AJ (2012) Altered placental methyl donor transport in the dexamethasone programmed rat. Placenta 33: 220–223. 22226642 10.1016/j.placenta.2011.12.017

[31] Weaver IC, Cervoni N, Champagne FA, D’Alessio AC, Sharma S, et al (2004) Epigenetic programming by maternal behavior. Nat Neurosci 7: 847–854. 15220929 10.1038/nn1276

[32] Oberlander TF, Weinberg J, Papsdorf M, Grunau R, Misri S, et al (2008) Prenatal exposure to maternal depression, neonatal methylation of human glucocorticoid receptor gene (NR3C1) and infant cortisol stress responses. Epigenetics 3: 97–106. 18536531 10.4161/epi.3.2.6034

[33] McGowan PO, Sasaki A, D’Alessio AC, Dymov S, Labonte B, et al (2009) Epigenetic regulation of the glucocorticoid receptor in human brain associates with childhood abuse. Nat Neurosci 12: 342–348. 19234457 10.1038/nn.2270 PMC2944040

[34] Perroud N, Paoloni-Giacobino A, Prada P, Olie E, Salzmann A, et al (2011) Increased methylation of glucocorticoid receptor gene (NR3C1) in adults with a history of childhood maltreatment: A link with the severity and type of trauma. Transl Psychiatry 1: e59. 22832351 10.1038/tp.2011.60 PMC3309499

[35] Radtke KM, Ruf M, Gunter HM, Dohrmann K, Schauer M, et al (2011) Transgenerational impact of intimate partner violence on methylation in the promoter of the glucocorticoid receptor. Transl Psychiatry 1: e21. 22832523 10.1038/tp.2011.21 PMC3309516

[36] Bromer C, Marsit CJ, Armstrong DA, Padbury JF, Lester B (2012) Genetic and epigenetic variation of the glucocorticoid receptor (NR3C1) in placenta and infant neurobehavior. Dev Psychobiol doi: 10.1002/dev.21061. 10.1002/dev.21061 PMC3458180 22714792

[37] Mulligan CJ, D’Errico NC, Stees J, Hughes DA (2012) Methylation changes at NR3C1 in newborns associate with maternal prenatal stress exposure and newborn birth weight. Epigenetics 7: 853–857. 22810058 10.4161/epi.21180 PMC3427280

[38] Alikhani-Koopaei R, Fouladkou F, Frey FJ, Frey BM (2004) Epigenetic regulation of 11 beta-hydroxysteroid dehydrogenase type 2 expression. J Clin Invest 114: 1146–1157. 15489962 10.1172/JCI21647 PMC522246

[39] Non AL, Binder AM, Barault L, Rancourt RC, Kubzansky LD, et al (2012) DNA methylation of stress-related genes and LINE-1 repetitive elements across the healthy human placenta. Placenta 33: 183–187. 22222044 10.1016/j.placenta.2011.12.013 PMC3308680

[40] Jensen Peña C, Monk C, Champagne FA (2012) Epigenetic effects of prenatal stress on 11beta-hydroxysteroid dehydrogenase-2 in the placenta and fetal brain. PLoS One 7: e39791. 22761903 10.1371/journal.pone.0039791 PMC3383683

[41] Jiang X, Yan J, West AA, Perry CA, Malysheva OV, et al (2012) Maternal choline intake alters the epigenetic state of fetal cortisol-regulating genes in humans. FASEB J 26: 3563–3574. 22549509 10.1096/fj.12-207894

[42] Yuen RK, Avila L, Peñaherrera MS, von Dadelszen P, Lefebvre L, et al (2009) Human placental-specific epipolymorphism and its association with adverse pregnancy outcomes. PLoS One 4: e7389. 19838307 10.1371/journal.pone.0007389 PMC2760756

[43] Yuen RK, Peñaherrera MS, von Dadelszen P, McFadden DE, Robinson WP (2010) DNA methylation profiling of human placentas reveals promoter hypomethylation of multiple genes in early-onset preeclampsia. Eur J Hum Genet 18: 1006–1012. 20442742 10.1038/ejhg.2010.63 PMC2987406

[44] Bourque DK, Avila L, Peñaherrera M, von Dadelszen P, Robinson WP (2010) Decreased placental methylation at the H19/IGF2 imprinting control region is associated with normotensive intrauterine growth restriction but not preeclampsia. Placenta 31: 197–202. 20060582 10.1016/j.placenta.2009.12.003

[45] Hogg K, Blair JD, von Dadelszen P, Robinson WP (2013) Hypomethylation of the LEP gene in placenta and elevated maternal leptin concentration in early onset pre-eclampsia. Mol Cell Endocrinol doi: 10.1016/j.mce.2012.12.018. 10.1016/j.mce.2012.12.018 23274423

[46] Magee LA, Helewa M, Moutquin JM, von Dadelszen P (2008) Hypertension Guideline Committee, et al (2008) Diagnosis, evaluation, and management of the hypertensive disorders of pregnancy. J Obstet Gynaecol Can 30: S1–48. 18817592 10.1016/S1701-2163(16)32776-1

[47] Kramer MS, Platt RW, Wen SW, Joseph KS, Allen A, et al (2001) A new and improved population-based canadian reference for birth weight for gestational age. Pediatrics 108: E35. 11483845 10.1542/peds.108.2.e35

[48] Du P, Zhang X, Huang CC, Jafari N, Kibbe WA, et al (2010) Comparison of beta-value and M-value methods for quantifying methylation levels by microarray analysis. BMC Bioinformatics 11: 587–2105-11-587. 21118553 10.1186/1471-2105-11-587 PMC3012676

[49] Maksimovic J, Gordon L, Oshlack A (2012) SWAN: Subset-quantile within array normalization for illumina infinium HumanMethylation450 BeadChips. Genome Biol 13: R44. 22703947 10.1186/gb-2012-13-6-r44 PMC3446316

[50] Price EM, Cotton AM, Lam LL, Farre P, Emberly E, et al.. (2013) Additional annotation enhances potential for biologically-relevant analysis of the illumina infinium Human Methylation 450 Bead Chip array. Epigenetics & Chromatin: In Press. 10.1186/1756-8935-6-4 PMC3740789 23452981

[51] Mikeska T, Felsberg J, Hewitt CA, Dobrovic A (2011) Analysing DNA methylation using bisulphite pyrosequencing. Methods Mol Biol 791: 33–53. 21913070 10.1007/978-1-61779-316-5_4

[52] Marsit CJ, Maccani MA, Padbury JF, Lester BM (2012) Placental 11-beta hydroxysteroid dehydrogenase methylation is associated with newborn growth and a measure of neurobehavioral outcome. PLoS One 7: e33794. 22432047 10.1371/journal.pone.0033794 PMC3303854

[53] Lee KJ, Shim SH, Kang KM, Kang JH, Park DY, et al (2010) Global gene expression changes induced in the human placenta during labor. Placenta 31: 698–704. 20554320 10.1016/j.placenta.2010.05.006

[54] Johnson RF, Rennie N, Murphy V, Zakar T, Clifton V, et al (2008) Expression of glucocorticoid receptor messenger ribonucleic acid transcripts in the human placenta at term. J Clin Endocrinol Metab 93: 4887–4893. 18728163 10.1210/jc.2008-1077

[55] Turner JD, Muller CP (2005) Structure of the glucocorticoid receptor (NR3C1) gene 5′ untranslated region: Identification, and tissue distribution of multiple new human exon 1. J Mol Endocrinol 35: 283–292. 16216909 10.1677/jme.1.01822

[56] Michael AE, Papageorghiou AT (2008) Potential significance of physiological and pharmacological glucocorticoids in early pregnancy. Hum Reprod Update 14: 497–517. 18552168 10.1093/humupd/dmn021

[57] Khulan B, Drake AJ (2012) Glucocorticoids as mediators of developmental programming effects. Best Pract Res Clin Endocrinol Metab 26: 689–700. 22980050 10.1016/j.beem.2012.03.007

[58] Cao-Lei L, Leija SC, Kumsta R, Wust S, Meyer J, et al (2011) Transcriptional control of the human glucocorticoid receptor: Identification and analysis of alternative promoter regions. Hum Genet 129: 533–543. 21234764 10.1007/s00439-011-0949-1

[59] Weaver IC, D’Alessio AC, Brown SE, Hellstrom IC, Dymov S, et al (2007) The transcription factor nerve growth factor-inducible protein a mediates epigenetic programming: Altering epigenetic marks by immediate-early genes. J Neurosci 27: 1756–1768. 17301183 10.1523/JNEUROSCI.4164-06.2007 PMC2951014

[60] Filiberto AC, Maccani MA, Koestler D, Wilhelm-Benartzi C, Avissar-Whiting M, et al (2011) Birthweight is associated with DNA promoter methylation of the glucocorticoid receptor in human placenta. Epigenetics 6: 566–572. 21521940 10.4161/epi.6.5.15236 PMC3121971

[61] Turner JD, Pelascini LP, Macedo JA, Muller CP (2008) Highly individual methylation patterns of alternative glucocorticoid receptor promoters suggest individualized epigenetic regulatory mechanisms. Nucleic Acids Res 36: 7207–7218. 19004867 10.1093/nar/gkn897 PMC2602793

[62] Avila L, Yuen RK, Diego-Alvarez D, Peñaherrera MS, Jiang R, et al (2010) Evaluating DNA methylation and gene expression variability in the human term placenta. Placenta 31: 1070–1077. 20947161 10.1016/j.placenta.2010.09.011

[63] Plotsky PM, Meaney MJ (1993) Early, postnatal experience alters hypothalamic corticotropin-releasing factor (CRF) mRNA, median eminence CRF content and stress-induced release in adult rats. Brain Res Mol Brain Res 18: 195–200. 8497182 10.1016/0169-328x(93)90189-v

[64] Takahashi LK, Turner JG, Kalin NH (1998) Prolonged stress-induced elevation in plasma corticosterone during pregnancy in the rat: Implications for prenatal stress studies. Psychoneuroendocrinology 23: 571–581. 9802128 10.1016/s0306-4530(98)00024-9

[65] Rots NY, de Jong J, Workel JO, Levine S, Cools AR, et al (1996) Neonatal maternally deprived rats have as adults elevated basal pituitary-adrenal activity and enhanced susceptibility to apomorphine. J Neuroendocrinol 8: 501–506. 8843018 10.1046/j.1365-2826.1996.04843.x

[66] Yang R, You X, Tang X, Gao L, Ni X (2006) Corticotropin-releasing hormone inhibits progesterone production in cultured human placental trophoblasts. J Mol Endocrinol 37: 533–540. 17170093 10.1677/jme.1.02119

[67] Goland RS, Conwell IM, Jozak S (1995) The effect of pre-eclampsia on human placental corticotrophin-releasing hormone content and processing. Placenta 16: 375–382. 7567800 10.1016/0143-4004(95)90095-0

[68] Goland RS, Tropper PJ, Warren WB, Stark RI, Jozak SM, et al (1995) Concentrations of corticotrophin-releasing hormone in the umbilical-cord blood of pregnancies complicated by pre-eclampsia. Reprod Fertil Dev 7: 1227–1230. 8848592 10.1071/rd9951227

[69] Ng EK, Leung TN, Tsui NB, Lau TK, Panesar NS, et al (2003) The concentration of circulating corticotropin-releasing hormone mRNA in maternal plasma is increased in preeclampsia. Clin Chem 49: 727–731. 12709362 10.1373/49.5.727

[70] Florio P, Imperatore A, Sanseverino F, Torricelli M, Reis FM, et al (2004) The measurement of maternal plasma corticotropin-releasing factor (CRF) and CRF-binding protein improves the early prediction of preeclampsia. J Clin Endocrinol Metab 89: 4673–4677. 15356079 10.1210/jc.2004-0186

[71] Galbiati S, Causarano V, Pinzani P, Francesca S, Orlando C, et al (2010) Evaluation of a panel of circulating DNA, RNA and protein potential markers for pathologies of pregnancy. Clin Chem Lab Med 48: 791–794. 20374043 10.1515/CCLM.2010.160

[72] Goland RS, Jozak S, Warren WB, Conwell IM, Stark RI, et al (1993) Elevated levels of umbilical cord plasma corticotropin-releasing hormone in growth-retarded fetuses. J Clin Endocrinol Metab 77: 1174–1179. 8077309 10.1210/jcem.77.5.8077309

[73] Karteris E, Goumenou A, Koumantakis E, Hillhouse EW, Grammatopoulos DK (2003) Reduced expression of corticotropin-releasing hormone receptor type-1 alpha in human preeclamptic and growth-restricted placentas. J Clin Endocrinol Metab 88: 363–370. 12519878 10.1210/jc.2002-020375

[74] Shenker N, Flanagan JM (2012) Intragenic DNA methylation: Implications of this epigenetic mechanism for cancer research. Br J Cancer 106: 248–253. 22166804 10.1038/bjc.2011.550 PMC3261681

[75] Mayor-Lynn K, Toloubeydokhti T, Cruz AC, Chegini N (2011) Expression profile of microRNAs and mRNAs in human placentas from pregnancies complicated by preeclampsia and preterm labor. Reprod Sci 18: 46–56. 21079238 10.1177/1933719110374115 PMC3343068

[76] Welberg LA, Seckl JR, Holmes MC (2000) Inhibition of 11beta-hydroxysteroid dehydrogenase, the foeto-placental barrier to maternal glucocorticoids, permanently programs amygdala GR mRNA expression and anxiety-like behaviour in the offspring. Eur J Neurosci 12: 1047–1054. 10762336 10.1046/j.1460-9568.2000.00958.x

[77] Strauss JF,3rd, Martinez F, Kiriakidou M (1996) Placental steroid hormone synthesis: Unique features and unanswered questions. Biol Reprod 54: 303–311. 8788180 10.1095/biolreprod54.2.303

[78] Watari H, Arakane F, Moog-Lutz C, Kallen CB, Tomasetto C, et al (1997) MLN64 contains a domain with homology to the steroidogenic acute regulatory protein (StAR) that stimulates steroidogenesis. Proc Natl Acad Sci U S A 94: 8462–8467. 9237999 10.1073/pnas.94.16.8462 PMC22957

[79] Tuckey RC, Bose HS, Czerwionka I, Miller WL (2004) Molten globule structure and steroidogenic activity of N-218 MLN64 in human placental mitochondria. Endocrinology 145: 1700–1707. 14715710 10.1210/en.2003-1034

[80] Ben-Zimra M, Koler M, Orly J (2002) Transcription of cholesterol side-chain cleavage cytochrome P450 in the placenta: Activating protein-2 assumes the role of steroidogenic factor-1 by binding to an overlapping promoter element. Mol Endocrinol 16: 1864–1880. 12145340 10.1210/me.2002-0056

[81] Hermann R, Doerfler W (1991) Interference with protein binding at AP2 sites by sequence-specific methylation in the late E2A promoter of adenovirus type 2 DNA. FEBS Lett 281: 191–195. 1826660 10.1016/0014-5793(91)80391-f

[82] Jacquemin P, Martial JA, Davidson I (1997) Human TEF-5 is preferentially expressed in placenta and binds to multiple functional elements of the human chorionic somatomammotropin-B gene enhancer. J Biol Chem 272: 12928–12937. 9148898 10.1074/jbc.272.20.12928

[83] Peng L, Huang Y, Jin F, Jiang SW, Payne AH (2004) Transcription enhancer factor-5 and a GATA-like protein determine placental-specific expression of the type I human 3beta-hydroxysteroid dehydrogenase gene, HSD3B1. Mol Endocrinol 18: 2049–2060. 15131259 10.1210/me.2004-0028 PMC3273420

[84] Bulun SE, Takayama K, Suzuki T, Sasano H, Yilmaz B, et al (2004) Organization of the human aromatase p450 (CYP19) gene. Semin Reprod Med 22: 5–9. 15083376 10.1055/s-2004-823022

[85] Mendelson CR, Kamat A (2007) Mechanisms in the regulation of aromatase in developing ovary and placenta. J Steroid Biochem Mol Biol 106: 62–70. 17596931 10.1016/j.jsbmb.2007.05.001 PMC2075360

[86] Demura M, Bulun SE (2008) CpG dinucleotide methylation of the CYP19 I.3/II promoter modulates cAMP-stimulated aromatase activity. Mol Cell Endocrinol 283: 127–132. 18201819 10.1016/j.mce.2007.12.003

[87] Knower KC, To SQ, Simpson ER, Clyne CD (2010) Epigenetic mechanisms regulating CYP19 transcription in human breast adipose fibroblasts. Mol Cell Endocrinol 321: 123–130. 20211687 10.1016/j.mce.2010.02.035

[88] Moldenhauer JS, Stanek J, Warshak C, Khoury J, Sibai B (2003) The frequency and severity of placental findings in women with preeclampsia are gestational age dependent. Am J Obstet Gynecol 189: 1173–1177. 14586374 10.1067/s0002-9378(03)00576-3

[89] Egbor M, Ansari T, Morris N, Green CJ, Sibbons PD (2006) Morphometric placental villous and vascular abnormalities in early- and late-onset pre-eclampsia with and without fetal growth restriction. BJOG 113: 580–589. 16579806 10.1111/j.1471-0528.2006.00882.x

[90] Goswami D, Tannetta DS, Magee LA, Fuchisawa A, Redman CW, et al (2006) Excess syncytiotrophoblast microparticle shedding is a feature of early-onset pre-eclampsia, but not normotensive intrauterine growth restriction. Placenta 27: 56–61. 16310038 10.1016/j.placenta.2004.11.007

[91] Rolfo A, Many A, Racano A, Tal R, Tagliaferro A, et al (2010) Abnormalities in oxygen sensing define early and late onset preeclampsia as distinct pathologies. PLoS One 5: e13288. 20967267 10.1371/journal.pone.0013288 PMC2953500

[92] van der Merwe JL, Hall DR, Wright C, Schubert P, Grove D (2010) Are early and late preeclampsia distinct subclasses of the disease–what does the placenta reveal? Hypertens Pregnancy 29: 457–467. 20701467 10.3109/10641950903572282

[93] Walsh SW (1988) Progesterone and estradiol production by normal and preeclamptic placentas. Obstet Gynecol 71: 222–226. 2962026

[94] Tamimi R, Lagiou P, Vatten LJ, Mucci L, Trichopoulos D, et al (2003) Pregnancy hormones, pre-eclampsia, and implications for breast cancer risk in the offspring. Cancer Epidemiol Biomarkers Prev 12: 647–650. 12869405

[95] Beaudoin C, Blomquist CH, Bonenfant M, Tremblay Y (1997) Expression of the genes for 3 beta-hydroxysteroid dehydrogenase type 1 and cytochrome P450scc during syncytium formation by human placental cytotrophoblast cells in culture and the regulation by progesterone and estradiol. J Endocrinol 154: 379–387. 9379114 10.1677/joe.0.1540379

