# Supplementary figures and images for: Early Onset Pre-Eclampsia Is Associated with Altered DNA Methylation of Cortisol-Signalling and Steroidogenic Genes in the Placenta
Source: PLoS One. 2013 May 7;8(5):e62969. doi: 10.1371/journal.pone.0062969 (PMC3647069; doi:10.1371/journal.pone.0062969)

**Figure S1**

**
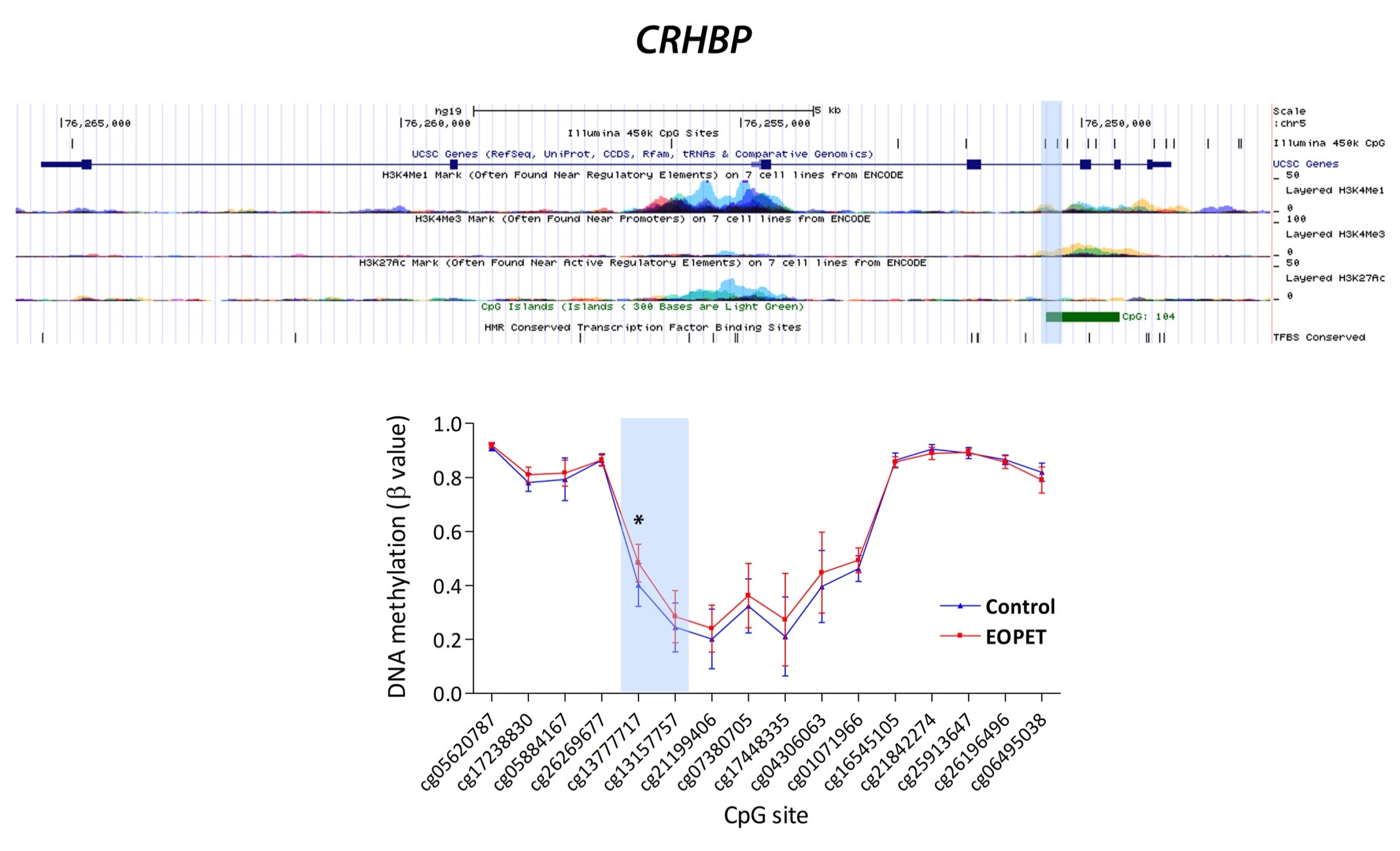
**

Supplement: Figure S1 — CpG sites on the Illumina Infinium HumanMethylation450 BeadChip array across the CRHBP gene region. Upper panel: UCSC genome browser shot detailing location of Illumina CpG probes, histone marks predictive of enhancers, CpG island(s) and conserved transcription factor binding sites (TFBS) associated with the gene body. Lower panel: DNA methylation pattern at Illumina CpG sites across the corticotropin releasing hormone binding protein (CRHBP) gene region in control (n = 19) and early onset pre-eclampsia (EOPET; n = 19) placentae. The Illumina CpG probe identifier is indicated by cg# and the position of CpG sites on the graphs are not to scale. Values are mean ± SD, and *P<0.05 based on Mann-Whitney tests. (DOCX) [file pone.0062969.s001.docx]

**Figure S2**


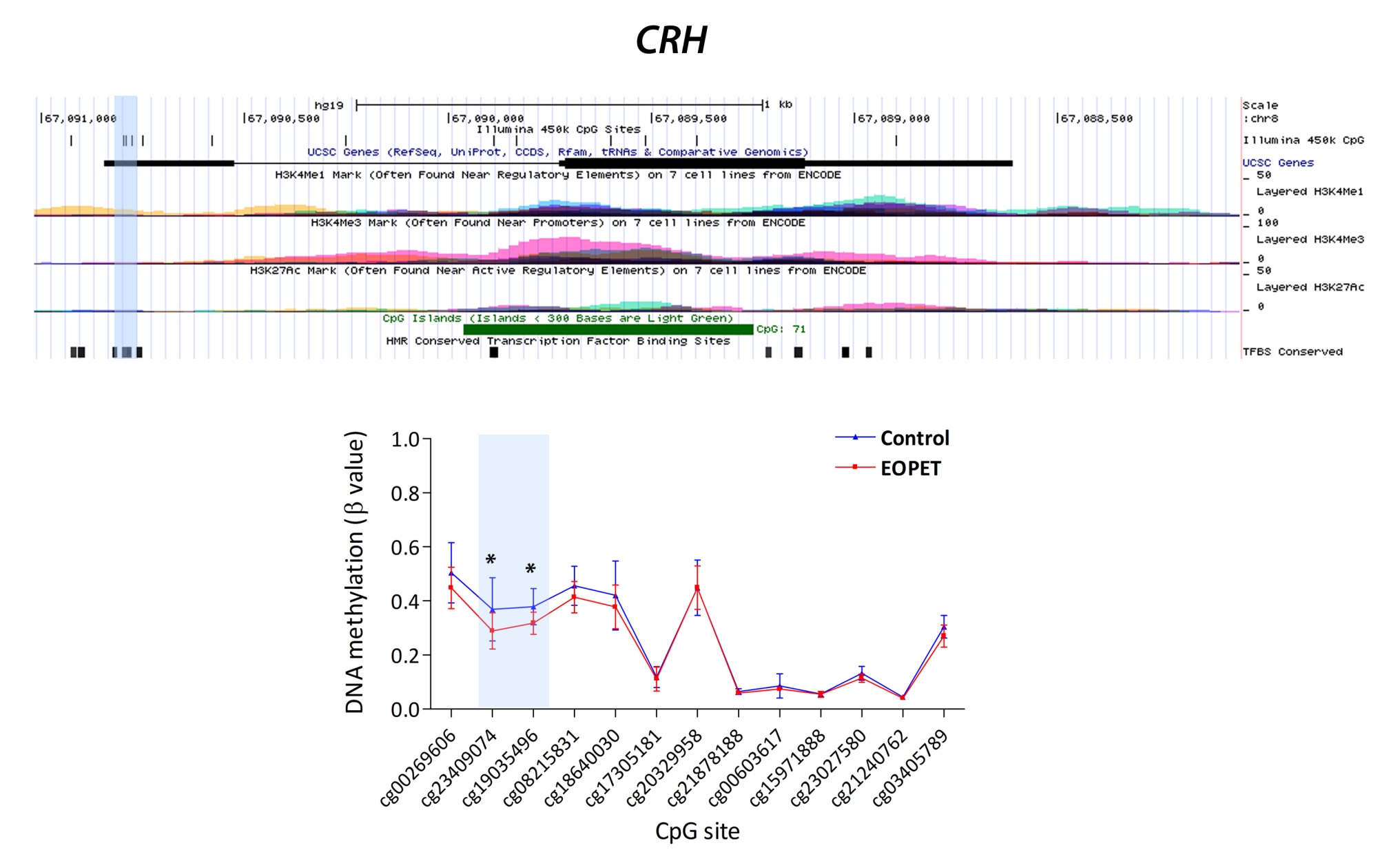

Supplement: Figure S2 — CpG sites on the Illumina Infinium HumanMethylation450 BeadChip array across the CRH gene region. Upper panel: UCSC genome browser shot detailing location of Illumina CpG probes, histone marks predictive of enhancers, CpG island(s) and conserved transcription factor binding sites (TFBS) associated with the gene body. Lower panel: DNA methylation pattern at Illumina CpG sites across the corticotropin releasing hormone (CRH) gene region in control (n = 19) and early onset pre-eclampsia (EOPET; n = 19) placentae. The Illumina CpG probe identifier is indicated by cg# and the position of CpG sites on the graphs are not to scale. Values are mean ± SD, and *P<0.05 based on Mann-Whitney tests. (DOCX) [file pone.0062969.s002.docx]

**Figure S3**


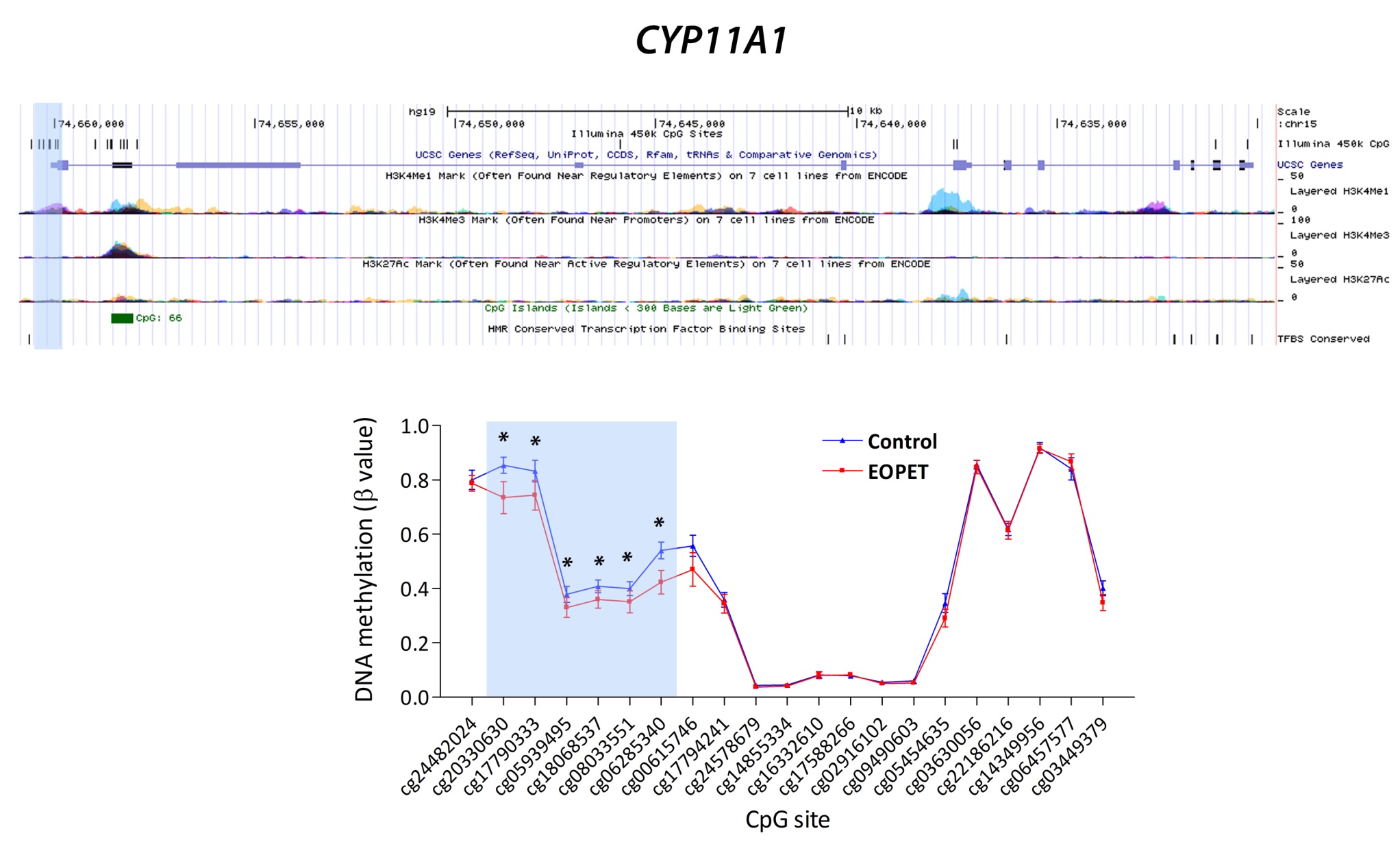

Supplement: Figure S3 — CpG sites on the Illumina Infinium HumanMethylation450 BeadChip array across the CYP11A1 gene region. Upper panel: UCSC genome browser shot detailing location of Illumina CpG probes, histone marks predictive of enhancers, CpG island(s) and conserved transcription factor binding sites (TFBS) associated with the gene body. Lower panel: DNA methylation pattern at Illumina CpG sites across the CYP11A1 gene region in control (n = 19) and early onset pre-eclampsia (EOPET; n = 19) placentae. The Illumina CpG probe identifier is indicated by cg# and the position of CpG sites on the graphs are not to scale. Values are mean ± SD, and *P<0.05 based on Mann-Whitney tests. (DOCX) [file pone.0062969.s003.docx]

**Figure S4**


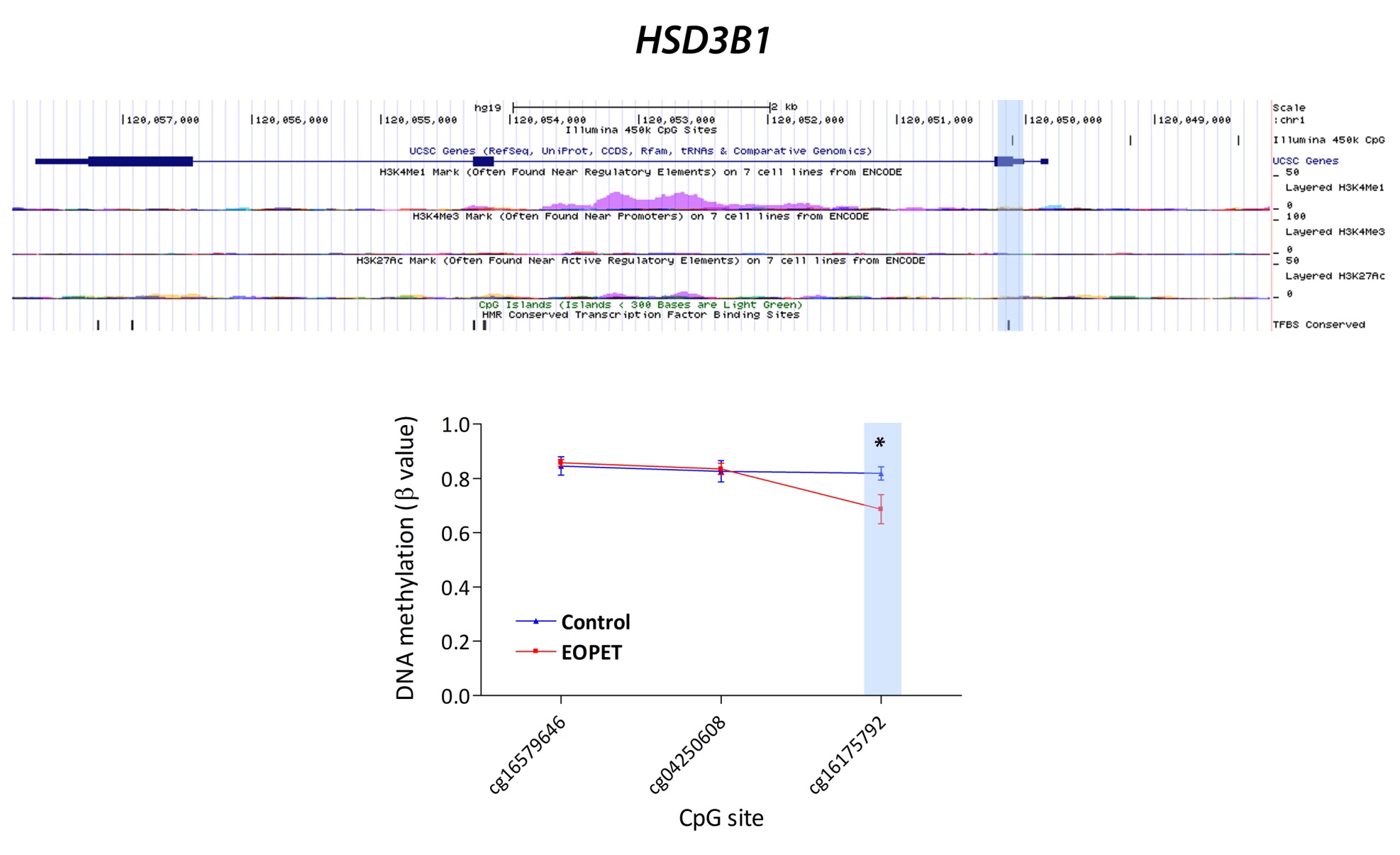

Supplement: Figure S4 — CpG sites on the Illumina Infinium HumanMethylation450 BeadChip array across the HSD3B1 gene region. Upper panel: UCSC genome browser shot detailing location of Illumina CpG probes, histone marks predictive of enhancers, CpG island(s) and conserved transcription factor binding sites (TFBS) associated with the gene body. Lower panel: DNA methylation pattern at Illumina CpG sites across the 3β-hydroxy-delta-5-steroid dehydrogenase type 1 (HSD3B1) gene region in control (n = 19) and early onset pre-eclampsia (EOPET; n = 19) placentae. The Illumina CpG probe identifier is indicated by cg# and the position of CpG sites on the graphs are not to scale. Values are mean ± SD, and *P<0.05 based on Mann-Whitney tests. (DOCX) [file pone.0062969.s004.docx]

**Figure S5**


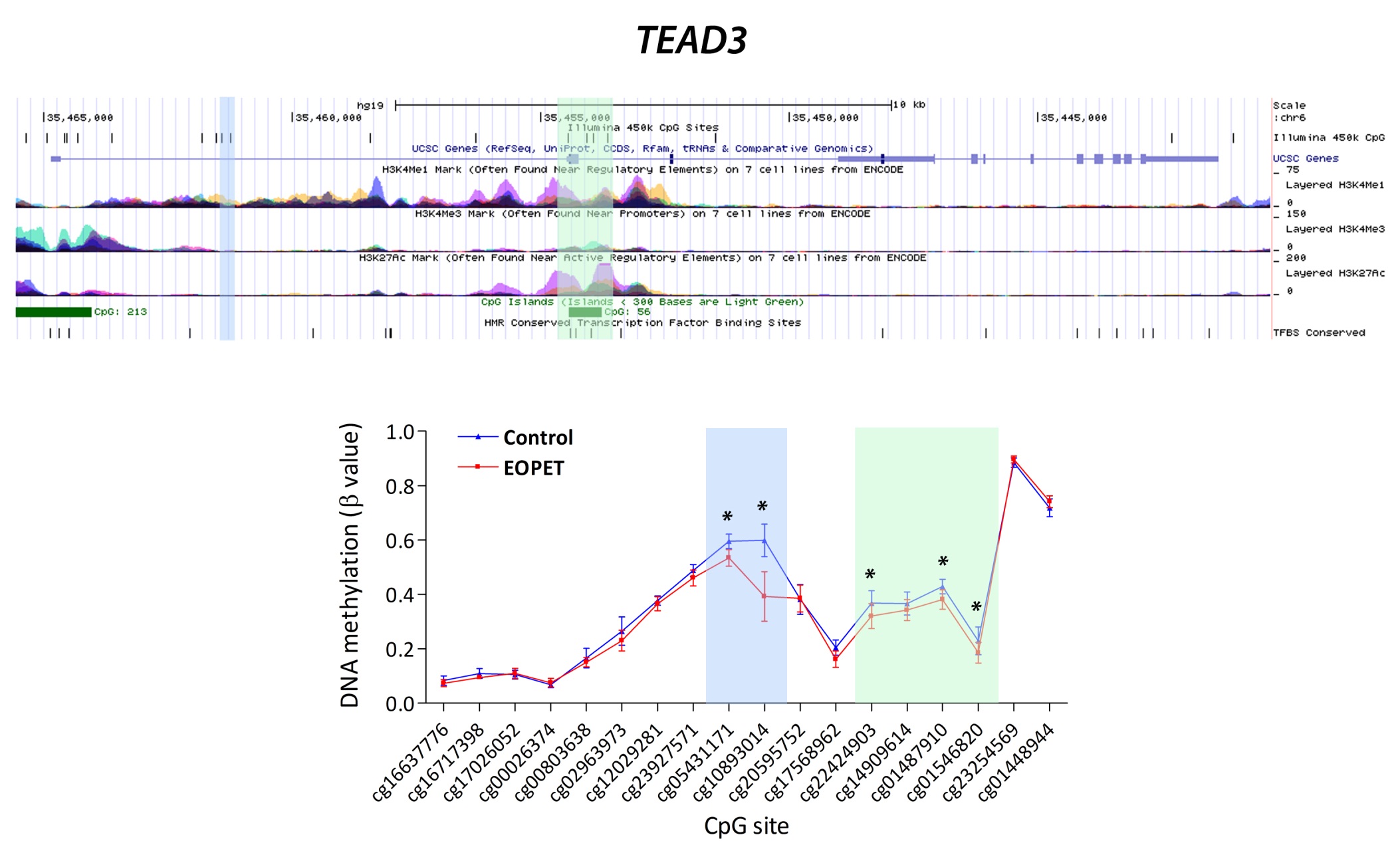

Supplement: Figure S5 — CpG sites on the Illumina Infinium HumanMethylation450 BeadChip array across the TEAD3 gene region. Upper panel: UCSC genome browser shot detailing location of Illumina CpG probes, histone marks predictive of enhancers, CpG island(s) and conserved transcription factor binding sites (TFBS) associated with the gene body. Lower panel: DNA methylation pattern at Illumina CpG sites across the TEA domain family member 3 (TEAD3) gene region in control (n = 19) and early onset pre-eclampsia (EOPET; n = 19) placentae. The Illumina CpG probe identifier is indicated by cg# and the position of CpG sites on the graphs are not to scale. Values are mean ± SD, and *P<0.05 based on Mann-Whitney tests. (DOCX) [file pone.0062969.s005.docx]

**Figure S6**


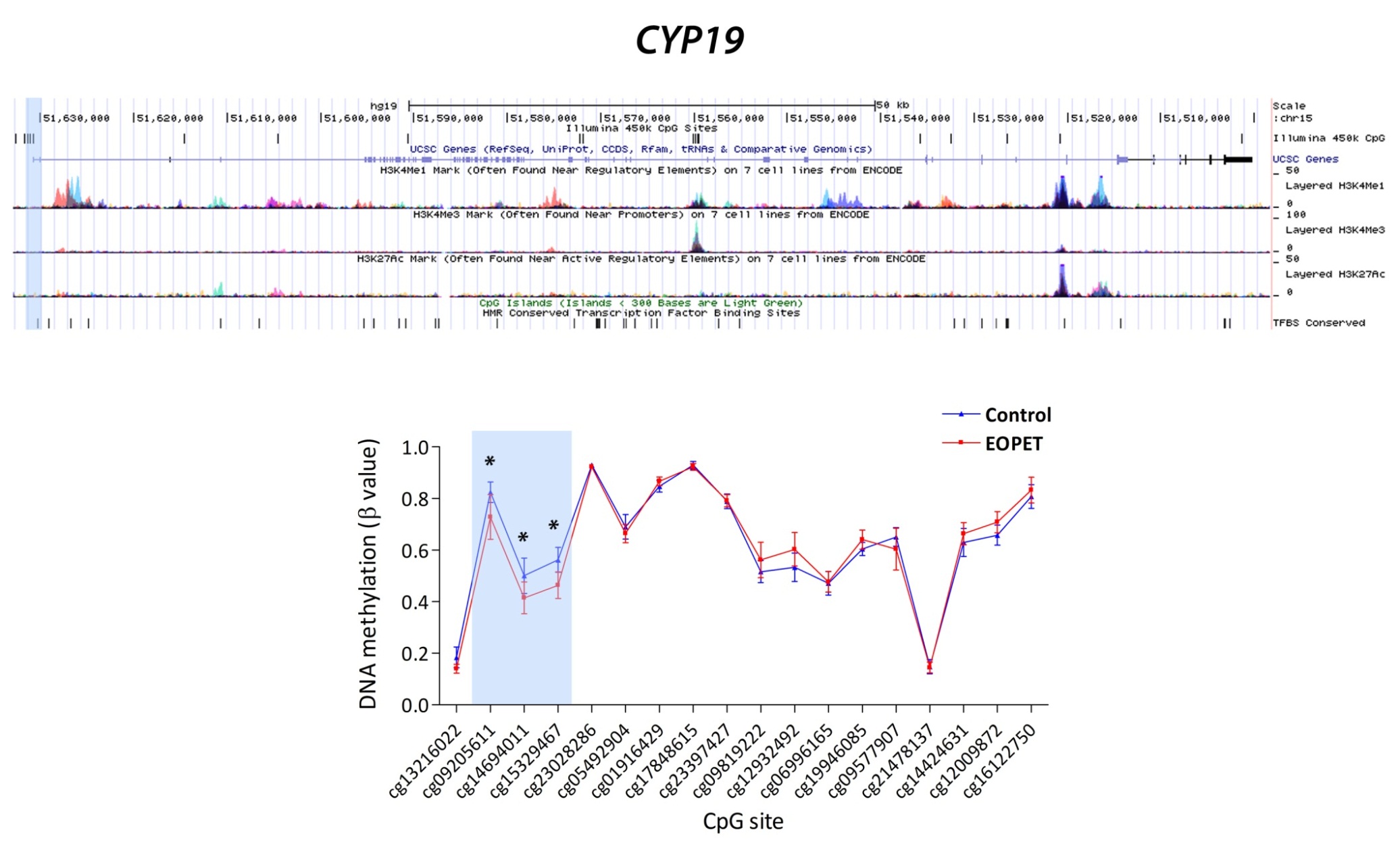

Supplement: Figure S6 — CpG sites on the Illumina Infinium HumanMethylation450 BeadChip array across the CYP19 gene region. Upper panel: UCSC genome browser shot detailing location of Illumina CpG probes, histone marks predictive of enhancers, CpG island(s) and conserved transcription factor binding sites (TFBS) associated with the gene body. Lower panel: DNA methylation pattern at Illumina CpG sites across the CYP19 gene region in control (n = 19) and early onset pre-eclampsia (EOPET; n = 19) placentae. The Illumina CpG probe identifier is indicated by cg# and the position of CpG sites on the graphs are not to scale. Values are mean ± SD, and *P<0.05 based on Mann-Whitney tests. (DOCX) [file pone.0062969.s006.docx]

**Figure S7**


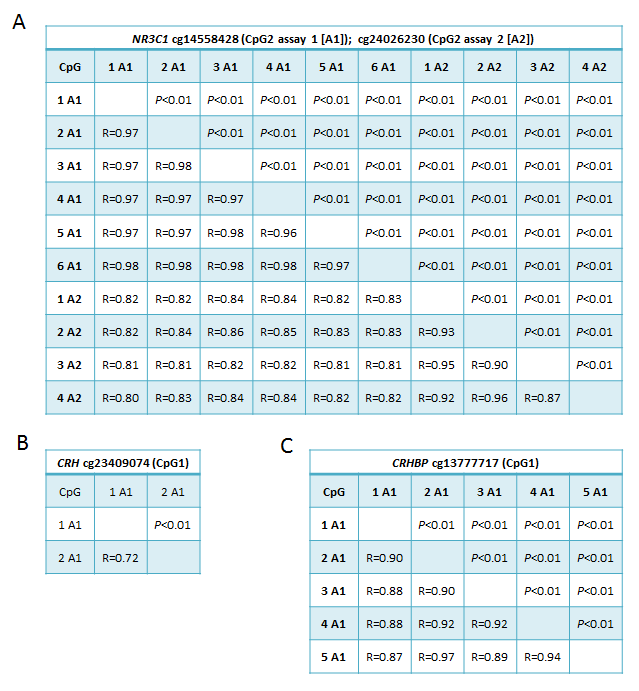


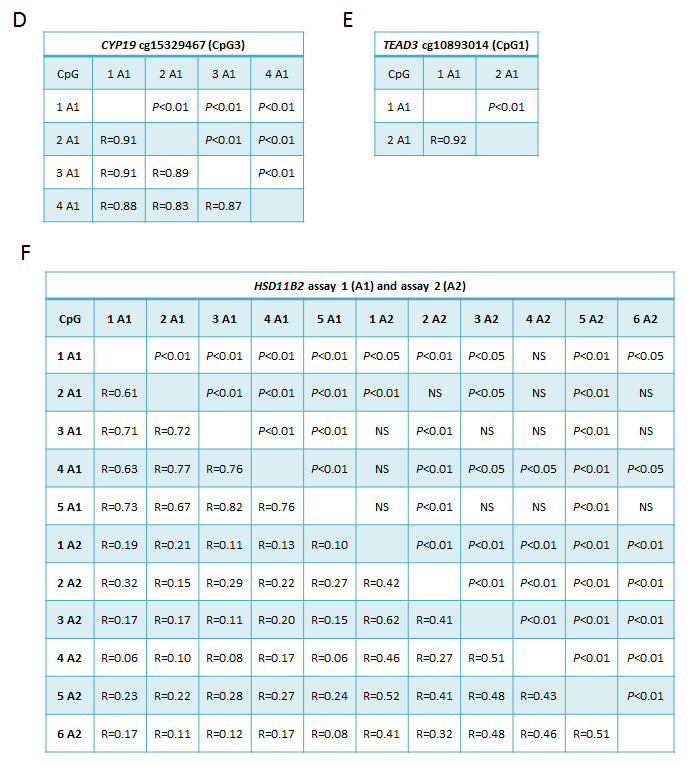
.

Supplement: Figure S7 — Assessment of assay CpG correlations where >1 CpG site was interrogated. A) nuclear receptor subfamily 3, group C, member 1 (NR3C1), B) corticotropin releasing hormone (CRH), C) CRH binding protein (CRHBP), D) CYP19, E) TEA domain family member 3 (TEAD3) and F) 11β-hydroxysteroid dehydrogenase type 2 (HSD11B2). Spearman Rho (R) values and corresponding P-values are given based on bisulfite pyrosequencing results. (DOCX) [file pone.0062969.s007.docx]
