# Supplementary material for: Early Onset Pre-Eclampsia Is Associated with Altered DNA Methylation of Cortisol-Signalling and Steroidogenic Genes in the Placenta
Source: PLoS One. 2013 May 7;8(5):e62969. doi: 10.1371/journal.pone.0062969 (PMC3647069; doi:10.1371/journal.pone.0062969)
Supplement: Table S1 — Primer sequences for bisulfite pyrosequencing assays. The genomic location of the PCR and individual CpG target, are based on Genome Browser hg19 version. F: forward, R: reverse, S: sequence, Bi: biotin label. NR3C1: nuclear receptor subfamily 3, group C, member 1, CRH: corticotropin releasing hormone, CRHBP: CRH binding protein: HSD3B1∶3β-hydroxy-delta-5-steroid dehydrogenase type 1, TEAD3: TEA domain family member 3, HSD11B1∶11β-hydroxysteroid dehydrogenase type 2. (DOCX) [file pone.0062969.s008.docx]

**Table S1**

| Gene | Primer | | Sequence (5'-3') | Genomic target (hg19) |
| --- | --- | --- | --- | --- |
| *NR3C1* Assay 1  NM_001204263 | F | TTATTTTTGAGAATTAAGGAAGG | | chr5: 142784928- 142785095 (168bp) |
|  | R | Bi- CCCCCTACTCTAACATCTTAAAA | |  |
| cg14558428 | S | ATATTGTATTTTATTAAGATGG | | 142784982 |
| *NR3C1* Assay 2  NM_001204263 | F | AGTTTGTTTTTTGGGTTTAGAAGG | | chr5: 142785132- 142785356 (225bp) |
|  | R | Bi-AAATAAACTTTCAACAAACCTCTT | |  |
| cg24026230 | S | GGTTTTAGAATTTTTTGGAG | | 142785172 |
| *CRH*  NM_000756 | F | TTGGTAGGGTTTTATGATTTATGT | | chr8: 67090658- 67090839 (182bp) |
|  | R | Bi-TTTCCCCTCAATCTCTCAATAAA | |  |
| cg23409074 | S | TTTATGTAGGAGTAGAGGTAGT | | 67090798 |
| *CRHBP*  NM_001882 | F | Bi-GAGTGTTGGGGTTTATTAAG | | chr5: 76250382- 76250584 (203bp) |
| cg13777717 | R | CCACCTATCTCAATCTAAATACC | | 76250527 |
|  | S | ATCCCCTACTACTCCAAAAT | |  |
| *CYP11A1*  NM_000781 | F | Bi-TGTGGGGATAGTATGTTGGTTAAGG | | chr15: 74659844- 74659938 (95bp) |
|  | R | CCCAACCCCTCCCTAAAAACACT | |  |
| cg06285340 | S | TAAAAACACTCAAAAAAATCT | | 74659901 |
| *HSD3B1*  NM_000862 | F | GGAAAATGAGGTATTTGTGTGAG | | chr1: 120049964- 120050143 (180bp) |
| cg16175792 | R | Bi-AAAAACCCTCCTACTCCTATCACA | | 120050104 |
|  | S | TGTTATTTTGGATGGTTATG | |  |
| *TEAD3*  NM_003214 | F | Bi-GTGTGTATTAGTAAGGTGGGTTT | | chr6: 35461155- 35461368 (214bp) |
| cg10893014 | R | CTACCTAACACAACCCTAAAAATA | | 35461236 |
|  | S | TCAAATAATAACCACCCACCCACA | |  |
| *CYP19*  NM_000103 | F | Bi-TTTTAGTTATGGATAAGAAATGAAATTT | | chr15:51630451- 51630851 (401bp) |
| cg15329467 | R | TATAATTCTTAAACCCTCCTATTTCAA | | 51630696 |
|  | S | ACAAATTAAAAACTCAATCAA | |  |
| *HSD11B2*  NM_000196 | F | TTAAGTTTTGGAAGGAAAGG | | chr16: 67464250-67464797 (365bp) |
|  | R | Bi-CTCAAATAAACACATACCACTCA | |  |
| Assay 1 | S | GGAAGGAAAGGGAAAGA | | 67464368-67464413 |
| Assay 2 | S | GTTAGTTTTTGTTTTAGGTA | | 67464443-67464499 |
