# Supplementary material for: Early Onset Pre-Eclampsia Is Associated with Altered DNA Methylation of Cortisol-Signalling and Steroidogenic Genes in the Placenta
Source: PLoS One. 2013 May 7;8(5):e62969. doi: 10.1371/journal.pone.0062969 (PMC3647069; doi:10.1371/journal.pone.0062969)
Supplement: Table S2 — HSD11B2 promoter methylation and infant birth weight. Univariate linear regression analyses for the relationship between birth weight (measured in standard deviation points relative to sex and age-matched normal birth weight ranges) and DNA methylation of CpG sites within the 11β-hydroxysteroid dehydrogenase type 2 (HSD11B2) promoter in placenta. (DOCX) [file pone.0062969.s009.docx]

**Table S2**

| CpG site | R | *P*-value |
| --- | --- | --- |
| 1 | 0.01 | 0.32 |
| 2 | 0.02 | 0.18 |
| 3 | 0.01 | 0.26 |
| 4 | 0.01 | 0.33 |
| 5 | 0.02 | 0.13 |
| 6 | <0.01 | 0.74 |
| 7 | <0.01 | 0.84 |
| 8 | <0.01 | 0.95 |
| 9 | <0.01 | 0.93 |
| 10 | 0.02 | 0.18 |
| 11 | <0.01 | 0.61 |
